# Physical cues influence the construction of the osteoimmune microenvironment: focus on the hematoma period

**DOI:** 10.1093/rb/rbag148

**Published:** 2026-08-05

**Authors:** Chenxi Fan, Lan Wang, Qinglian Yang, Yidan Wang, Zhengquan Wang, Wenhao Zhou, Pingyun Yuan, Mi Chen, Tian Bai, Xiaocheng Li, Lei Luo, Sen Yu

**Affiliations:** College of Metallurgical Engineering, Xi’an University of Architecture and Technology, Xi’an 710055, China; Shaanxi Key Laboratory of Biomedical Metallic Materials, Northwest Institute for Non-ferrous Metal Research, Xi’an 710016, China; Shaanxi Key Laboratory of Biomedical Metallic Materials, Northwest Institute for Non-ferrous Metal Research, Xi’an 710016, China; Shaanxi Key Laboratory of Biomedical Metallic Materials, Northwest Institute for Non-ferrous Metal Research, Xi’an 710016, China; Shaanxi Key Laboratory of Biomedical Metallic Materials, Northwest Institute for Non-ferrous Metal Research, Xi’an 710016, China; Shaanxi Key Laboratory of Biomedical Metallic Materials, Northwest Institute for Non-ferrous Metal Research, Xi’an 710016, China; Shaanxi Key Laboratory of Biomedical Metallic Materials, Northwest Institute for Non-ferrous Metal Research, Xi’an 710016, China; Shaanxi Key Laboratory of Biomedical Metallic Materials, Northwest Institute for Non-ferrous Metal Research, Xi’an 710016, China; Shaanxi Key Laboratory of Biomedical Metallic Materials, Northwest Institute for Non-ferrous Metal Research, Xi’an 710016, China; Shaanxi Key Laboratory of Biomedical Metallic Materials, Northwest Institute for Non-ferrous Metal Research, Xi’an 710016, China; Shaanxi Key Laboratory of Biomedical Metallic Materials, Northwest Institute for Non-ferrous Metal Research, Xi’an 710016, China; College of Metallurgical Engineering, Xi’an University of Architecture and Technology, Xi’an 710055, China; Shaanxi Key Laboratory of Biomedical Metallic Materials, Northwest Institute for Non-ferrous Metal Research, Xi’an 710016, China

**Keywords:** implant surface, immunomodulation, hematoma, immune cell

## Abstract

Hematoma formation represents one of the earliest biological events during bone healing after bone injury or biomaterial implantation, a process that involves coagulation, provisional matrix formation, innate immune activation and early adaptive immune signaling. During this stage, the hematoma is rapidly infiltrated by neutrophils, monocytes/macrophages, dendritic cells and lymphocytes, which collectively construct the initial osteoimmune microenvironment that modulates subsequent angiogenesis, osteogenesis and osseointegration. Therefore, a systematic elucidation of the hematoma formation process and the regulatory factors governing hematoma behavior provides an important foundation for designing immunomodulatory bone implants. However, most current research has focused primarily on the modulation of macrophages by surface characteristics, relying heavily on simplified *in vitro* monoculture systems, with limited *in vivo* temporal characterization of the intact hematoma microenvironment. Insufficient investigation of other immune cells and the regulation of hematomas limits our in-depth understanding of the interplay between hematomas and immunity. This review focuses on the hematoma as a spatiotemporally regulated immune niche and summarizes how material-derived biophysical cues, including surface topography, porosity, wettability and mechanical stiffness, may influence hematoma evolution and the downstream establishment of the osteoimmune microenvironment. This review also discusses the current challenges and future research directions in unraveling the complex material–hematoma–immunity axis, aiming to provide a conceptual framework for the development of translationally relevant immunomodulatory biomaterials for both physiological and pathological bone repair.

## Graphical abstract

**Graphical Abstract:**
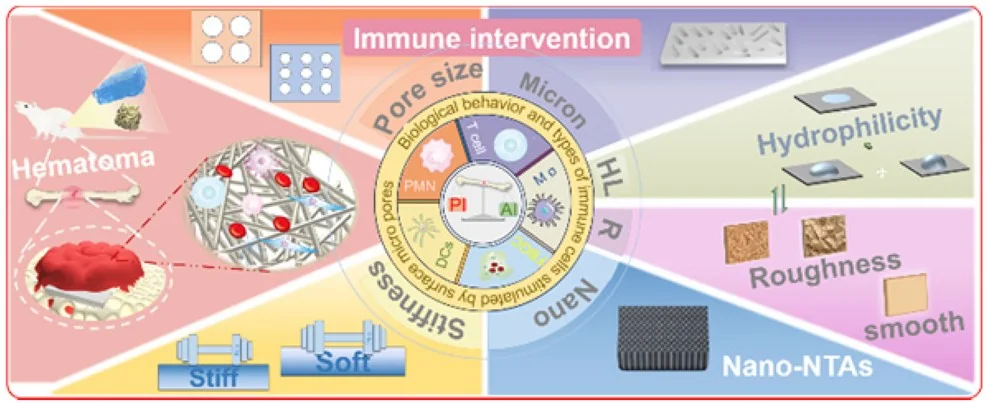

## Introduction

Bone healing is a complex and dynamic biological process modulated by the coordinated interplay of multiple cellular and molecular signaling pathways. Histologically, this process is generally categorized into four consecutive phases: hematoma formation/inflammation, soft callus formation, hard callus formation and bone remodeling [1]. The emergence of osteoimmunology has substantially reshaped our understanding of bone regeneration, demonstrating that bone healing is regulated not merely by the balance between bone resorption and bone formation, but also by reciprocal interactions between the skeletal and immune systems [2, 3]. Recent advances in bone biology and enabling technologies have further reinforced this evolving concept, which has deepened mechanistic insight into cell–cell communication, spatiotemporal tissue dynamics and the regulation of regeneration at multiple scales [4]. Immune regulation is initiated immediately after bone injury, where in the initial hematoma acts as the primary site of early host response. Upon biomaterial implantation, innate and adaptive immune responses synergize to establish a peri-implant immune microenvironment, which subsequently modulates the behavior of surrounding stromal, vascular and skeletal cells [5].

In the context of implantation, the hematoma constitutes the earliest biological interface between the biomaterials and host tissues [6]. Rather than being a simple by-product of injury, the hematoma functions as a dynamic provisional niche consisting of fibrin matrices, adsorbed proteins, infiltrating immune cells and recruited progenitor cells [7]. Accumulating evidence reveals that implant surface properties can remodel this early niche by modulating immune cell recruitment, adhesion, activation and phenotypic polarization, thereby influencing the subsequent balance of inflammation resolution, osseointegration and fibrotic encapsulation [8, 9]. Thus, deciphering the interactions between implant surfaces and hematoma-associated cells is very valuable for the rational design of osteoimmunomodulatory biomaterials [10, 11].

Despite the rapid expansion of osteoimmunology-oriented implant research, the hematoma phase remains an underexplored, stage-specific regulatory window. In particular, how early infiltrating immune cells sense and respond to implant-derived physical cues has not been fully elucidated. Meanwhile, implant surface design has evolved from simply promoting osteogenesis to actively shaping a pro-regenerative osteoimmune microenvironment. In-depth understanding of hematoma formation and its immune regulation is therefore important to guide the rational development of biomaterial surfaces. In this review, we summarize the modulation of the immune microenvironment by material surface properties, with a specific focus on immune cell behavior during the hematoma phase. We first illustrate the temporal dynamics of the major cell populations associated with the hematoma and the regulation of the hematoma architecture by the implant surface, and then discuss the regulatory effects of the implant surface on immune cell behavior. This review aims to synthesize current evidence and highlight key knowledge gaps in this research field.

## The hematoma: a spatiotemporal orchestrator of bone regeneration

### Temporal evolution of hematoma-stage immune responses

The hematoma formed upon implantation is not a static blood clot, but a dynamic and temporally organized biological entity that modulates the earliest stages of bone healing. Immediately after implantation, surgically induced tissue injury and vascular disruption trigger hematoma formation, thereby initiating the host response at the blood–material interface [12]. Structurally and biologically, the hematoma serves as a fibrin-rich provisional matrix containing entrapped erythrocytes, activated platelets, thrombin, adsorbed plasma proteins, and complement components. This view is supported by the work of Kolar *et al.*, who conducted a systematic review on fracture hematoma. They reported that it constitutes a transient tissue enriched in platelets, leukocytes and erythrocytes, rather than a simple passive coagulum. Importantly, they also demonstrated that the hematoma microenvironment is hypoxic and abundant in growth factors such as transforming growth factor beta 1 (TGF-β1), platelet-derived growth factor (PDGF) and vascular endothelial growth factor (VEGF). This microenvironment thus provides essential early cues for immune activation, angiogenesis and subsequent tissue regeneration [13]. Within the initial minutes to hours, the hematoma acts as the primary structural and biochemical platform for blood–material interactions, protein adsorption and fibrin assembly. Over the following hours to days, it functions as a transient scaffold supporting the rapid infiltration of innate immune cells, particularly neutrophils and monocytes. As the earliest recruited immune cells, neutrophils mediate antimicrobial defense, debris clearance and cytokine secretion, while also facilitating the formation of neutrophil extracellular traps (NETs) and provisional matrix remodeling. These functions collectively modulate subsequent macrophage recruitment and polarization. Monocytes subsequently differentiate into macrophages and DCs, which further participate in inflammatory amplification, antigen presentation and the transition toward tissue repair. At later stages, adaptive immune cells, including T cells and B lymphocytes, are progressively recruited and function to regulate inflammation and subsequent bone remodeling. Days later, the hematoma is gradually replaced by granulation tissue, laying a foundation for angiogenesis, osteogenesis and callus formation. To fully understand bone healing progression, time-resolved profiling of biological events within the hematoma microenvironment is indispensable. Accordingly, we illustrate the dynamic evolution of the hematoma and summarize immune cell populations present at each stage.

#### Hematoma formation (minutes to hours)

Upon implantation, plasma proteins rapidly adsorb onto material surfaces, promoting platelet adhesion and activation. This biological cascade ultimately drives the formation of a fibrin-based hematoma surrounding the implant [14]. The composition of the hematoma, particularly thrombin and fibrin content, together with the physicochemical properties of the implant surface, exerts profound effects on the subsequent inflammatory response [15]. Romero *et al.* [16], through precise temporal analysis of protein adsorption on titanium surfaces (2, 180 and 960 min), reported that calcium-rich surfaces enriched coagulation-related proteins (e.g. Factor XII, Factor XI) and complement proteins as early as 2 min, thereby inducing a pro-coagulant response and initiating early inflammatory signaling. Li *et al.* [17] further reported that superhydrophilic surfaces with vertically oriented mesoporous silica films tended to promote the formation of a denser fibrin network within minutes by preferentially accumulating acidic proteins and coagulation factors, promoting platelet activation and hematoma formation. Kenny *et al.* [18] reported that nanofibrous topography is an important regulatory factor in modulating the interaction between platelets and fibrinogen, suggesting the regulatory capacity of morphology on hematoma-related processes. Lackington *et al.* systematically characterized blood–material interactions on three titanium surfaces (smooth hydrophobic, rough hydrophobic and rough hydrophilic nanostructured). Their results demonstrated that rough surfaces stimulated a denser fibrin network, while hydrophilic surfaces accelerated the coagulation rate [19]. In summary, rapid protein adsorption, platelet activation and fibrin assembly represent the earliest events at the blood–material interface and shape the initial architecture of the peri-implant hematoma. Cumulative evidence indicates that surface chemistry, hydrophilicity and topography can modulate these early processes by altering adsorbed protein composition and fibrin organization. However, most studies adopt simplified *in vitro* systems, making it challenging to dissect the independent effects of individual surface parameters. Moreover, direct links between early clot architecture and long-term regenerative outcomes remain poorly defined. Future studies may benefit from integrating time-resolved hematoma characterization with clinically relevant *in vivo* models.

#### Hematoma maturation (hours to days)

Schmidt-Bleek *et al.* [20] defined the hematoma as the ‘initial template’ for tissue regeneration and further delineated the kinetics of immune cell infiltration. Within 6–24 h after injury, the hematoma is established and rapidly infiltrated by neutrophils [21, 22]. These early responders subsequently recruit monocytes, which differentiate into macrophages within the subsequent 48–96 h [20, 23].

**Figure 1:**
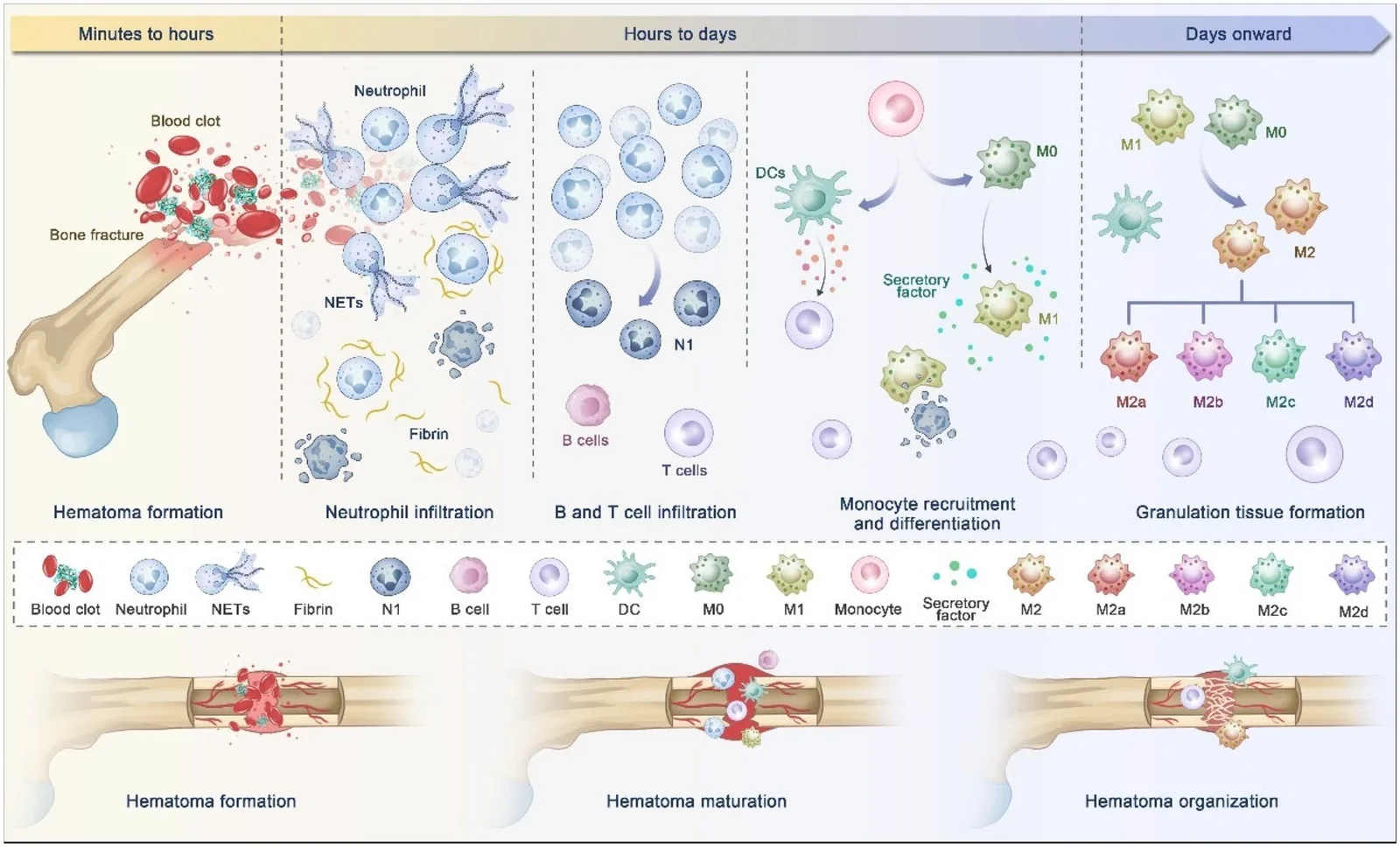
The hematoma evolves as a dynamic, time-resolved biological entity that modulates subsequent bone regeneration. Hematoma formation (minutes to hours): rapid protein adsorption, coagulation cascade activation, complement deposition and fibrin network formation establish the initial hematoma architecture and set the stage for immune cell recruitment. Hematoma maturation (hours to days): neutrophils infiltrate (peaking at 6–24 h) and release NETs; monocytes are recruited (48–96 h) and differentiate into dendritic cells (DCs) and macrophages; T cells and B cells appear within 24–72 h. Hematoma organization (days onward): macrophages transition from a pro-inflammatory M1 to a pro-regenerative M2 phenotype, adaptive immunity becomes more prominent, and the hematoma is gradually replaced by granulation tissue, marking the shift from inflammation to bone regeneration.

As illustrated in Figure 1, neutrophils, as the first responders, rapidly infiltrate the implantation site during the acute inflammatory phase [13], critically modulating the recruitment of macrophages and other immune cells to the injured microenvironment [24]. Neutrophils exhibit a spectrum of pro-inflammatory and anti-inflammatory phenotypes and possess functional plasticity beyond phagocytosis, although the mechanisms are less defined than those of macrophages. By secreting mediators such as IL-1β, CXCL1/3, TNF-α and myeloperoxidase-derived reactive oxygen species, neutrophils regulate subsequent immune responses, recruiting and polarizing infiltrating macrophages [25]. This early event exerts a considerable and long-lasting influence on the downstream biological cascade and long-term implant osseointegration outcomes, with its effects closely related to the intensity and duration of cell activation. Neutrophil adhesion behavior is primarily governed by early plasma protein adsorption [26]. By altering fibrinogen adsorption, molecular conformation and fibril organization, surface chemical properties modulate fibrinogen biological activity, which further dictates subsequent neutrophil behaviors [27].

The intensity and duration of neutrophil activation are important modulators of bone healing efficiency and trajectory. Excessive or persistent activation, especially under pathological hyper-inflammatory states, may lead to aberrant neutrophil infiltration and excessive NET release, ultimately compromising bone regeneration. NET formation is an important defense mechanism of neutrophils, and these web-like structures, composed of decondensed chromatin, myeloperoxidase and neutrophil elastase, play pivotal roles in modulating neutrophil-mediated immunity [28, 29]. *In vitro* studies have demonstrated that excessive neutrophil accumulation suppresses the function of bone marrow mesenchymal stem cells (BMSCs), reducing cell proliferation, alkaline phosphatase activity and mineralized extracellular matrix (ECM) synthesis [30]. In a tendon-bone injury model, Zhou *et al.* systematically elucidated the mechanism by which early NETs accumulation aggravates healing impairment via modulating macrophage polarization. They revealed that rapid neutrophil infiltration and NET release significantly upregulated M1 markers while downregulating M2 markers. In contrast, inhibition of NET formation reversed this phenotypic shift and alleviated early peritendinous fibrosis [31]. Although this study focused on tendon-bone healing, this mechanistic insight into NET-mediated macrophage regulation may provide valuable insights for understanding the hematoma phase. Pathological conditions can further exacerbate the negative impact of NETs. Zhao *et al.* found that obese mice induced by a high-fat diet exhibited earlier neutrophil infiltration and increased NET formation during the endochondral ossification phase of fracture healing, resulting in delayed bone repair. Mechanistically, NETs inhibited BMSC osteogenic differentiation and promoted M1 macrophage polarization by activating the NLRP3 inflammasome, suggesting that targeting NETs may be a strategy for treating obesity-related delayed fracture healing [32]. Neutrophils are not merely an immune ‘first responder’, but also an active contributor to early matrix formation. Within 48 h after fracture, they constitute the major cellular population at the injury site. In addition to their phagocytic activity, hematoma-associated neutrophils facilitate the synthesis of fibronectin-containing ECM, thereby promoting the transition of the hematoma into reparative granulation tissue [33].

Similar to M1-like macrophages, neutrophils primarily rely on glycolytic metabolism, which enables their adaptation to the hypoxic microenvironment of the hematoma. Pro-inflammatory N1 neutrophils may be directly recruited by IL-17 or arise from undifferentiated neutrophils recruited by TNF-α, acting in concert with M1-like macrophages and secreting mediators such as IFN-γ, IL-6, IL-12, IL-14, IL-16, IL-18, IL-19, CCL2 and CCL20 [34]. Nevertheless, the functional contribution of individual neutrophil-derived cytokine pathways may be context-dependent. For example, Fischer *et al.* [35] showed in neutrophil-specific IL-6 or IL-6R knockout mice that loss of neutrophil-derived IL-6 signaling exerts negligible effects on fracture healing outcomes. Consistent with this view, temporal analysis of human fracture hematomas further suggested phenotype-associated neutrophil heterogeneity, with Cathepsin D (CTSD) enriched in pro-regenerative N2-like neutrophils and Cathepsin G (CTSG) enriched in pro-inflammatory N1-like neutrophils [36]. These findings highlight the complexity and systemic plasticity of neutrophil-mediated immunomodulation in bone repair.

Following the initial neutrophil infiltration, monocytes are recruited and differentiate into DCs and macrophages. DCs appear in the early stages of fracture healing and express inflammatory cytokines (IL-6, IL-12, TNF-α, IL-10) [37, 38], playing an important regulatory role in subsequent healing phases. The number of DCs peaks during the inflammatory phase and returns to baseline during callus formation and remodeling [39, 40]. DCs are notable participants in the early immune response to implants and serve as an important bridge between innate and adaptive immunity. As professional antigen-presenting cells, they contribute to shaping the osteoimmune microenvironment. Functionally, similar to macrophages, DCs have a dual role during the healing process by promoting initial inflammation while facilitating the resolution of inflammation at later stages. Upon contact with blood or tissue fluid, macromolecules such as proteins rapidly adsorb onto the biomaterial surface. These adsorbed molecules, whether exogenous antigens or self-antigens, can be recognized by DCs or internalized, processed and presented to initiate an adaptive immune response.

Classically activated M1 macrophages, defined by surface markers CCR7, CD80/86 and NOS2, exert a dual regulatory role during the early hematoma stage. On the one hand, M1 macrophages promote hematoma remodeling by phagocytosing fibrin clots, necrotic debris and apoptotic neutrophils, accelerating fibroblast-mediated collagen deposition and granulation tissue formation to replace the initial hematoma. On the other hand, they secrete a spectrum of pro-inflammatory cytokines and chemokines (including IL-6, CCL2, IL-1, IL-17, IFN-γ and TNF-α) to amplify local inflammation, promote osteoclast differentiation and activation and facilitate local bone resorption [41]. Despite their critical role in debris clearance and inflammation initiation, excessive M1 activation can cause damage to surrounding healthy bone tissue. Notably, the M1 response can be modulated by the implant’s surface properties. Moderate surface-mediated M1 activation can create a pro-regenerative microenvironment that favors mesenchymal stem cell (MSC) osteogenesis, while activated macrophages secrete TNF-α to initiate crucial angiogenesis [42, 43]. The dynamic switching of macrophages between M1 and M2 phenotypes plays an important role in bone regeneration. The M1/M2 ratio, critical for initiating and resolving inflammation, needs to be tightly regulated, as premature switching to M2 phenotype can lead to insufficient inflammation and healing disorders [44].

Notably, T cells massively infiltrate within 24–72 h post-injury, persisting through the callus formation phase. *In vivo* animal studies have shown that T cells are visible throughout the fracture hematoma area, from surrounding muscles to the fracture gap, as early as 3 days post-fracture [45, 46]. Furthermore, murine fracture models revealed that skeletal injury significantly elevated T cell proportions in the bone marrow of contralateral limbs within 24 h, and this elevation persisted for 21 days, underscoring the substantial impact of fracture on T cell homeostasis. Interestingly, one study found that during the inflammatory phase, gut-derived T helper 17 (Th17) cells were recruited to the fracture site to promote healing, whereas γδ T cells expanded in the callus of murine femurs on day 1 post-fracture, thereby inducing systemic inflammation and increasing gut permeability [47]. Although T cell participation in the inflammatory phase is well established, the precise role and function of these cells in the hematoma stage remain uncertain.

During hematoma maturation, B cells and T cells infiltrate synchronously, peaking early (1–3 days) and participating in bone remodeling later. Notably, B cells retreat from fracture sites as acute inflammation resolves but reaccumulate robustly at day 14 post-injury during callus mineralization. Existing studies suggest that B cells undergo effector cell maturation during healing, secreting high levels of osteoclastogenesis inhibitory factor (OPG), which indicates a regulatory role during the bone remodeling phase [48, 49]. This separation of early presence and late function suggests that B cells may primarily act as a ‘sentinel’ in the hematoma phase, preparing for later functions through local cytokine secretion or antigen presentation, although the underlying mechanisms remain to be elucidated.

Accumulating evidence has revised the traditional view of adaptive immune activation timing, demonstrating that adaptive immune cell involvement occurs much earlier than previously recognized during hematoma development. Fischer *et al.* [50] employed AI-assisted single-cell clustering analysis and identified multiple B and T cell subsets in the fracture hematoma as early as day 1 post-injury, although their functional significance remains to be elucidated. These early-appearing adaptive immune cells may influence the subsequent immune response through local cytokine secretion or interaction with antigen-presenting cells. This is consistent with the observation by Könnecke *et al.* [48], who reported massive B cell infiltration on day 14 post-injury to exert regulatory functions. Notably, how material-derived physical cues modulate the recruitment and functional behavior of T and B cells during the hematoma phase remains largely unexplored.

#### Hematoma organization (days onward)

As the hematoma evolves over the following days, the initial fibrin-rich inflammatory clot is gradually remodeled and replaced by granulation tissue, accompanied by ECM reorganization, vascular ingrowth and partial resolution of acute inflammation. Within this shifting microenvironment, macrophages undergo phenotypic switching from a pro-inflammatory M1 state toward a more pro-regenerative M2 phenotype [51, 52]. M2 macrophages are heterogeneous and can be subdivided into M2a, M2b, M2c and M2d subtypes.

The M2a subtype, activated by IL-4 or IL-13, suppresses pro-inflammatory cytokine secretion (e.g. IL-1β, IL-6, TNF-α) and promotes tissue repair by enhancing angiogenesis, fibroblast proliferation and migration, and direct secretion of fibrogenic proteins to drive collagen deposition. The M2b phenotype is a regulatory phenotype that balances anti- and pro-inflammatory functions, characterized by high IL-10 and low IL-12 secretion. The M2c subtype, induced by IL-10, glucocorticoids, or TGF-β, further suppresses inflammation by downregulating pro-inflammatory cytokine expression, promoting debris clearance and endothelial cell proliferation/migration at the injury site, thereby playing a notable role in angiogenesis [53]. The M2d macrophage, generated via co-stimulation of Toll-like receptors (TLRs) and adenosine A2A receptors with their corresponding agonists, is characterized by high secretion of IL-10 and VEGF, and low secretion of TNF-α and IL-12 [54].

Collectively, M2 macrophages mitigate inflammation via inhibitory factors such as IL-1RA, IL-10 and TGF-β. Notably, IL-10 and IL-1RA exert bone-protective effects, as IL-10 can directly inhibit osteoclast function. Furthermore, M2 macrophages secrete TGF-β1 and TGF-β3, which suppress inflammation and initiate bone regeneration, partly through the regulation of fibrous capsule formation [55]. An M2-dominant microenvironment supports chondrogenic differentiation of stem cells at the injury site, promoting endochondral ossification. Woven bone is subsequently deposited, gradually enveloping the cartilage callus and providing mechanical stability. The established vasculature within the soft callus further promotes its transition to a hard callus. Healing then progresses to the remodeling phase, in which outer woven bone is gradually replaced by lamellar bone, internal callus and cartilage matrix are resorbed by osteoclasts, and bone tissue gradually regains its physiological structure and function [1].

Hoff *et al.* profiled cytokine and chemokine concentrations in 42 human long bone fracture samples at 72 h post-trauma. Proteomic analysis revealed that the hematoma was enriched with growth factors such as TGF-β1, PDGF and VEGF, as well as pro-inflammatory cytokines like IL-6 and IL-8, suggesting that the hematoma possesses both pro-inflammatory and pro-regenerative microenvironmental characteristics [56]. Wiesli *et al.* constructed a hematoma-mimicking microenvironment by co-culturing collagen-hydroxyapatite scaffolds with human whole blood, in which the blank scaffolds induced obvious upregulation of pro-inflammatory factors (IL-6, IL-8), resulting in impaired osteogenic capacity of progenitor cells. However, scaffolds loaded with BMP-7 and IL-10 markedly downregulated these inflammatory mediators and established a microenvironment more conducive to matrix mineralization [57]. The study indicates the potential of immunomodulatory strategies in an *in vitro* hematoma-mimicking microenvironment, providing new insights for the development of hematoma-targeted therapeutic interventions.

### Hematoma architecture influences healing outcomes

The formation of a stable hematoma is correlated with successful osseointegration. The hematoma not only provides mechanical stabilization at the fracture site but also serves as a bioactive reservoir of platelets and leukocytes [58]. Cellular composition analysis reveals distinct differences between the hematoma and peripheral blood, characterized by elevated granulocyte-to-lymphocyte and monocyte-to-lymphocyte ratios [59]. Furthermore, fracture hematomas are enriched with anti-inflammatory mediators that modulate osteoclastogenesis, stimulate angiogenesis, and facilitate ECM synthesis. Pountos *et al.* [60] validated these observations through clinical analysis of 23 patient samples, confirming that fracture hematomas enhance osteogenic differentiation, as evidenced by upregulated alkaline phosphatase activity and calcium deposition. The absence of a stable hematoma is correlated with an elevated risk of delayed bone healing or non-union, whereas the hematoma at the injury site establishes the early healing microenvironment that lays the biological foundation for subsequent bone regeneration [61].

This understanding has inspired the construction of ‘biomimetic hematomas’, namely engineered scaffolds integrating key cellular components of whole blood (erythrocytes, platelets, leukocytes and osteoprogenitors) [62, 63]. These structures provide vital regulatory signals for osteoblast differentiation and bone formation, exhibiting promising translational potential for repairing large segmental bone defects [64].

The quality of the initial hematoma affects early healing outcomes, with the fibrin clot structure directly guiding the cell infiltration pattern required for bone regeneration [65, 66]. Specifically, the 3D fibrin network regulates key biological processes, including cell migration and cytokine signaling, and may affect bone repair efficiency. Woloszyk *et al.* demonstrated a correlation between hematoma microstructure and bone healing. In different bone defect models (normal healing, delayed healing, non-union), obvious differences were observed in the fibrin characteristics (diameter, density, porosity, stiffness) of the hematoma. Furthermore, RNA sequencing identified 700 differentially expressed genes. These findings indicate that hematoma structure is closely associated with subsequent healing trajectories [67]. Biomaterials can modulate hematoma properties, promoting the assembly of dense, regularly organized fibrin networks with enhanced fibrinolytic resistance. As reported by Xiao *et al.* [68], a fibrin matrix with optimized thickness and density is conducive to bone regeneration. The ‘ideal’ hematoma structure observed in spontaneously healing defects provides a natural blueprint for successful regeneration, inspiring the design of a new generation of biomaterial-hematoma composite implants [69].

### Implant surface and hematoma immune modulation

Cumulative evidence demonstrates that surface microtopography, particularly nanostructures, serves as a key regulator of early hematoma formation. For example, 15 nm titanium dioxide nanotube arrays (TN-15) attenuate platelet activation and facilitate the formation of a dynamically suitable, loosely structured fibrin network within the hematoma. The TN-15 nanotube arrays are also associated with increased expression of pro-regenerative growth factors and M2-related macrophage markers, consequently promoting a more favorable osteoimmune microenvironment [70]. Furthermore, material properties, including surface roughness and wettability, regulate early blood–implant interfacial interactions. Although coagulation occurs on both microrough hydrophobic and hydrophilic titanium surfaces, hydrophilic surfaces tend to promote the formation of a dense, continuous fibrin network, whereas hydrophobic surfaces mostly produce isolated fibers and scattered patches [71]. Subsequent studies reported that the denser fibrin structure on hydrophilic substrates enhanced cell recruitment [72].

Collectively, these findings illustrate that implant surface properties can dictate early hematoma formation and organization, thereby orchestrating the establishment of a pro-osteogenic immunomodulatory microenvironment. Accordingly, the design of biomedical implants increasingly emphasizes physical parameters such as macro-/micro-architecture, hydrophilicity, and roughness to modulate the early post-implantation microenvironment [73–75]. By utilizing these biophysical cues during the hematoma phase, material surfaces can guide the immune responses toward promoting regeneration while minimizing the detrimental inflammatory response.

In summary, the immunological characteristics of the hematoma phase are largely modulated by the physicochemical properties of the implant surface. However, the specific mechanisms by which physical properties (e.g. roughness, hydrophilicity, nanostructure) influence plasma protein conformation, platelet activation, fibrin network architecture and subsequent immune cell behavior via micro- and nanoscale interfacial interactions remain to be clarified. Building on this foundation, ‘Physical regulation of the osteoimmune microenvironment’ section systematically elaborates how material surface topography, porosity, pore size, hydrophilicity, roughness and mechanical stiffness regulate the polarization, migration behavior and functional phenotypes of key immune cells, including macrophages, neutrophils and DCs. By establishing a relationship model of ‘material-hematoma-immunity’, this review aims to provide theoretical guidance for designing the next generation of immunomodulatory biomaterials.

## Physical regulation of the osteoimmune microenvironment

As discussed in the previous section, the hematoma phase constitutes the earliest stage of peri-implant healing and governs the recruitment and organization of immune cells at the implant interface. During this period, the local immune response evolves from an acute inflammatory phase dominated by innate immune cells toward a more coordinated reparative and adaptive immune environment, thereby exerting long-term effects on osseointegration. In addition to these hematoma-associated processes, implant surface properties can also regulate immune-cell behavior by influencing protein adsorption, fibrin organization, cell adhesion and biophysical signaling. Accordingly, this section comprehensively summarizes how key physical parameters, including topography, porosity, pore size, hydrophilicity, roughness, stiffness and hardness, modulate macrophages, T cells, DCs and neutrophils. The primary objective is to integrate general principles of osteoimmunology with the hematoma-driven evolution of the osteoimmune microenvironment.

### Surface topography

Surface micro-/nano-topography can influence hematoma organization by modulating early events such as protein adsorption, platelet adhesion and fibrin assembly at the blood–material interface. These early changes are important because they shape the provisional hematoma matrix and thereby affect subsequent immune-cell recruitment, adhesion, polarization and function. Beyond these initial blood-mediated effects, surface topographical features also directly modulate immune cell morphology, cytoskeletal rearrangement, mechanotransduction, and intercellular communication. Importantly, the immunomodulatory effects of surface topography are not confined to a specific cell type or single healing phase, but act across multiple stages of tissue repair from early hematoma organization to subsequent inflammatory resolution and pro-regenerative signal transduction. Herein, we systematically review the modulatory effects of micro-/nano-topography on the functional behaviors of key immune cell populations during bone repair, with a particular emphasis on topography-guided osteoimmune regulation within the early hematoma microenvironment.

#### Macrophages

**Figure 2:**
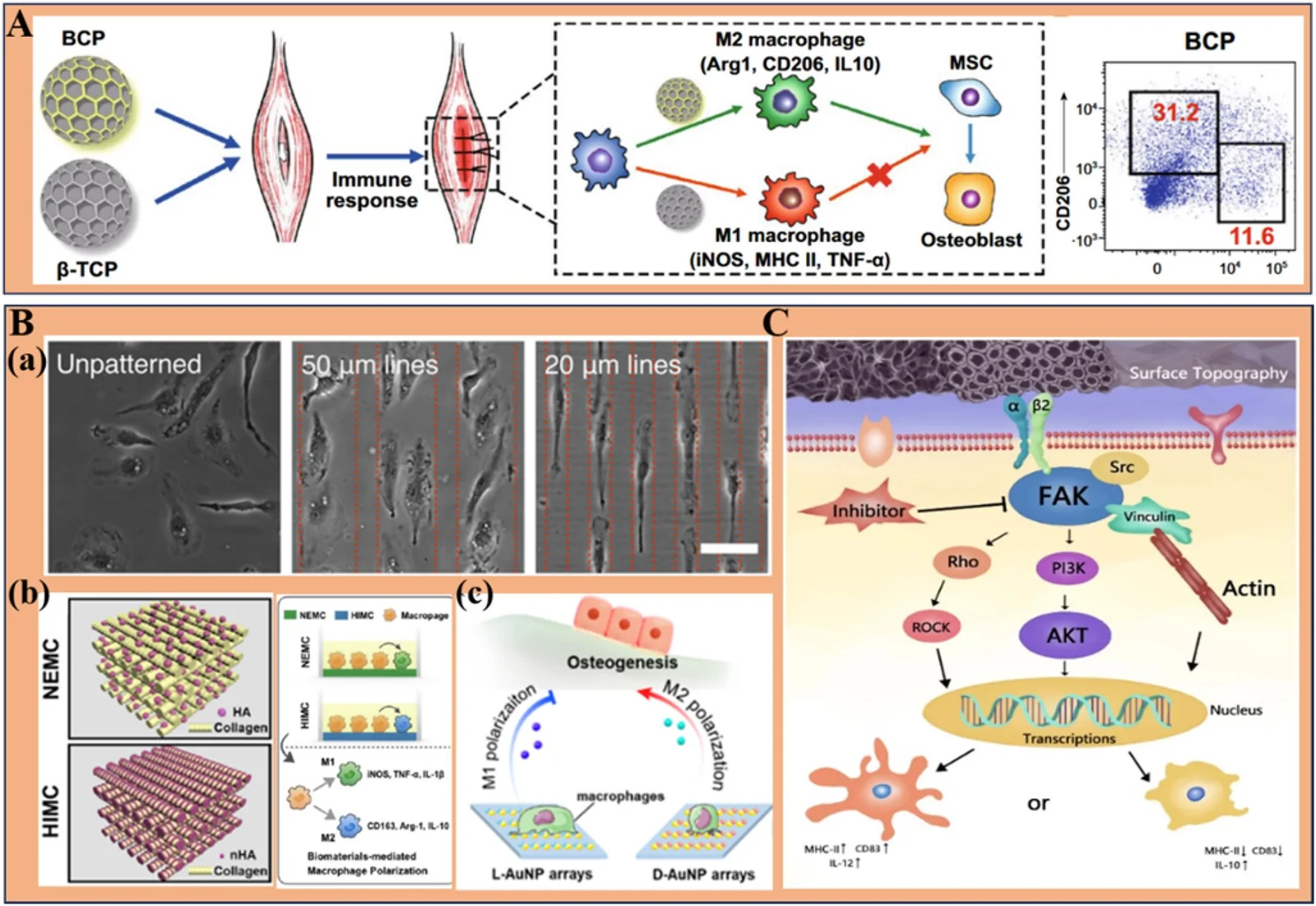
Macrophage responses under micro-nano structures. (**A**) Schematic diagram of biomaterial-mediated macrophage polarization and MSC osteogenesis. Reproduced with permission from Ref. [94], Copyright 2021, Springer. (**B**) (a) Effect of macrophage morphology on polarization. Reproduced with permission from Ref. [77], Copyright 2013, PNAS. (b) Effect of hierarchical micro- and nanostructures on macrophage polarization. Reproduced with permission from Ref. [84], Copyright 2019, ACS. (c) Effect of surface structure chirality on macrophage polarization. Reproduced with permission from Ref. [86], Copyright 2023, Wiley. (**C**) Micro- and nanostructures influence the cell adhesion pathway of DCs. Reproduced with permission from Ref. [93], Copyright 2022, Taylor & Francis.

Macrophage polarization is profoundly modulated by surface topography through the regulation of cell adhesion, spreading, elongation and migration. For example, Yang *et al.* [76] reported that micro-/nano-gridded titanium surfaces drove an elongated macrophage morphology through the Src–ROCK signaling pathway, which correlated with upregulated expression of M2 macrophage markers *in vitro*. Importantly, this morphology-dependent immunomodulation is not limited to rigid metallic substrates but also extends to low-stiffness materials, as observed by McWhorter *et al*. [77], who reported consistent macrophage elongation on low-stiffness fibronectin stripe-patterned substrates even without exogenous cytokine induction (Figure 2B-a). Notably, this topography-mediated immune response driven by morphological remodeling has been validated across diverse biomaterial systems. Yan *et al.* [78] fabricated graphene microgroove arrays and found that the microgrooves facilitated macrophage elongation rather than a spherical morphology, while suppressing the secretion of the M1-associated cytokine TNF-α *in vitro*. These findings indicate that macrophage polarization is modulated by cell morphology, which is transduced intracellularly via cytoskeletal rearrangement.

Nano-scale surface topography exerts size-dependent effects on macrophage-mediated immune responses, with appropriately sized nano-topographical features tending to favor an anti-inflammatory macrophage phenotype. Liang *et al.* [79] prepared nanotopographic titanium coatings and reported that surfaces with 180 nm plate-like structures induced activation of both M1 and M2 macrophage phenotypes, whereas larger plate-like features (440 and 780 nm) drove upregulated expression of M1 macrophage markers. Similarly, Chen *et al.* [80] observed that increasing the height or depth of nanostructures from 10 to 100 nm promoted higher production of pro-inflammatory factors. This size-dependent pro-inflammatory tendency was further verified in nanogrooved substrate studies, in which nanostructures with larger groove depths (32.7–158 nm) and ridge widths (71.9–536.4 nm) readily fostered a pro-inflammatory microenvironment [81].

**Figure 3:**
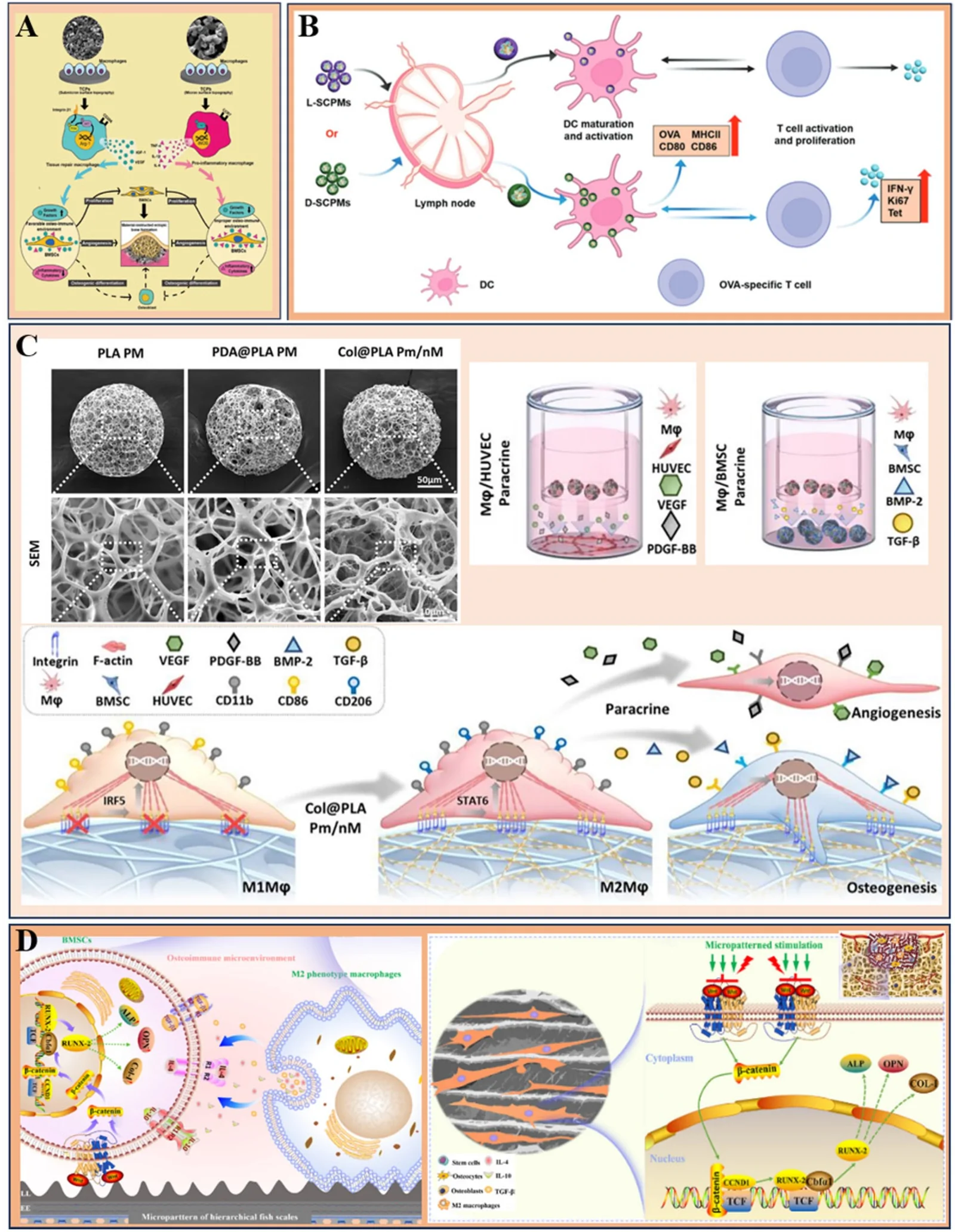
Effects of micro-implanted material structures and biomimetic structures on immune cell mechanisms. (**A**) Effect of particle size on macrophage polarization. Reproduced with permission from Ref. [82], Copyright 2020, RSC. (**B**) Schematic illustrations of the preparation of SCPMs and chirality-mediated antigen-specific immune activation. Reproduced with permission from Ref. [92], Copyright 2022, Wiley. (**C**) Col@PLA Pm/nM regulating Mφ, BMSCs/HUVECs activities, paracrine secretion and initiation of cascade signaling to reprogram the immune microenvironment for angiogenesis and bone repair. Reproduced with permission from Ref. [87], Copyright 2022, Elsevier. (D) Schematic illustration of the fish scales topological ridge micropatterned surface mediating the osteogenic differentiation of rBMSCs via the Wnt/β-catenin pathway. Reproduced with permission from Ref. [88], Copyright 2023, Elsevier.

Beyond nanoscale regulation, microscale surface features also contribute to the regulation of macrophage inflammatory phenotype, with a more pronounced size-dependent pro-inflammatory trend. For instance, Li *et al.* [82] used submicron-scale (TCPs) and micron-scale (TCPb) tricalcium phosphate ceramics to confirm this size-dependent immunomodulatory effect, finding that macrophages cultured on submicron tricalcium phosphate surfaces tended to polarize toward an M2-like phenotype, whereas those in the micron-scale group exhibited a pro-inflammatory M1-like state (Figure 3A). Likewise, Mestres *et al.* compared the immunomodulatory effects of nano- and microstructured hydroxyapatite substrates and reported distinct macrophage responses to crystal morphology. Macrophages cultured on microscale substrates produced higher levels of ROS, whereas ROS release on nanoscale substrates was minimal. These findings demonstrate that microscale topography favors macrophage pro-inflammatory activation, while nanostructures promote low-inflammatory or more M2 (anti-inflammatory) polarization [83].

On this basis, hierarchical micro-/nano-scale structural design enables topography-dependent osteoimmune microenvironment remodeling via precise regulation of macrophage behaviors. Jin *et al.* developed bone-mimetic hierarchically intrafibrillarly mineralized collagen (HIMC), which guided macrophages to align along the fibrils and induced IL-4-mediated M2 (anti-inflammatory) polarization. In contrast, the nano-structured NEMC containing spherical hydroxyapatite clusters triggered altered macrophage adhesive morphology and enhanced M1 polarization (Figure 2B-b) [84]. In another example, Bai *et al.* [85] constructed a biomimetic micro-/nano-topographic fibrous TiO_2_ network (MNS) on titanium implants, which favored M2 macrophage polarization. Collectively, these studies suggest that hierarchical micro-/nano-scale design can complement the functions of single-scale topography, providing a promising strategy for constructing an anti-inflammatory osteoimmune microenvironment via synergistic modulation of macrophage adhesion and polarization.

Macrophage polarization is governed not only by structural dimensionality but also by specific geometric configurations and spatial arrangements of surface topography. To further explore structure-mediated immune regulation, He *et al.* [86] fabricated chiral gold nanorod arrays and found that right-handed (D-type) nanostructures enhanced activation of the integrin αv/α8/β3–phosphorylated FAK signaling pathway in macrophages, thereby driving M2 polarization and increasing the secretion of anti-inflammatory cytokines (Figure 2B-c). As shown in Figure 3C, related studies have further employed hierarchical micro-/nano-topographies on nano-implants, in which massive collagen nanofibers were deposited in a spider web-like manner onto microsphere scaffold surfaces. This strategy established a stable and uniform gradient micro-/nano-topography both inside and outside the pores, which could mimic the ECM, activate integrin-mediated macrophage polarization, achieve paracrine-mediated microenvironmental reprogramming and ultimately drive macrophage polarization toward M2 phenotype [87]. In summary, three-dimensional topography also represents a valuable approach for regulating macrophages to generate a favorable immune microenvironment.

In terms of structural optimization, biomimetic structural design is a noteworthy research direction. Specifically, biomimetic strategies that replicate the surface topography of natural biological tissues have emerged as promising approaches for immunomodulation. Inspired by the structure of fish scales, Qin *et al.* fabricated ridge-like microstructures that markedly promoted macrophage polarization toward M2 phenotype (Figure 3D). By faithfully recapitulating native tissue structural cues, such biomimetic designs can effectively establish an osteoimmune microenvironment conducive to tissue regeneration, thereby underscoring the clinical translational value of biomimetic structural design [88].

Taken together, macrophages are highly sensitive to the structural dimensions of implant surfaces and exhibit distinct phenotypic responses to specific topographical cues. Microscale structures are predominantly associated with cell spreading and M1-like activation, whereas nanoscale features tend to favor M2-like polarization. In addition, hierarchical micro-/nano-architectures may integrate these complementary effects to generate a pro-regenerative immune microenvironment. Importantly, the benefits of topographical design extend beyond the regulation of macrophage polarization. Recent studies suggest that lamellar nanostructures and hybrid zinc-based nanostructures can simultaneously support both antibacterial activity and osteogenesis [89, 90], highlighting the need for optimizing physical surface cues within a multifunctional framework.

#### T cells

Exploring the impact of surface morphological changes on T cell responses typically requires integration with other cell behaviors. Song *et al.* reported that nanotopographical cues guide endothelial cell alignment and consequently modulate T cell transendothelial migration (TEM), a critical step in leukocyte infiltration. By culturing endothelial cells on nano-ridge/groove-patterned surfaces, highly aligned endothelial monolayers were generated. T cells migrating across these aligned endothelial layers exhibited reduced migration speeds and frequencies compared with cells traversing randomly oriented endothelial monolayers on flat surfaces [91]. In parallel, chiral nanostructures can drive modest antigen-specific T cell responses by modulating DC function, while also mitigating the cytotoxicity of cationic polymers. Specifically, D-chiral configurations exhibited minimal cytotoxicity, facilitated DC antigen uptake and maturation, and upregulated co-stimulatory molecule expression (Figure 3B). This ultimately contributed to enhanced proliferation of the antigen-specific T cells in the spleen, lymph nodes and tumor tissues of mice [92].

#### Dendritic cells

DCs also undergo substantial phenotypic transformation in response to micro-/nano-topographic cues on material surfaces. Yang *et al.* cultured DCs on different titanium surfaces and observed distinct morphological adaptations. On smooth surfaces (SA), DCs displayed a stellate or irregular shape with prominent branching, whereas on nanostructured (SAN) and microstructured (SAA) surfaces, they maintained a more rounded morphology with reduced branching (Figure 2C). The expression of the anti-inflammatory cytokine IL-10 was the lowest in the SA group and the highest in the SAA group. Significant differences in the β2 integrin–FAK signaling pathway were detected at both 4 and 24 h, suggesting that micro-/nano-topography is associated with the modulation of DC adhesion, morphology, gene expression and inflammatory status [93].

As a key mediator bridging innate and adaptive immunity, DCs are responsive to the combined effects of calcium and phosphate ion release and micro-/nano-structured surface topography of biomaterials. Zhao *et al.* reported that the micro-/nano-rough surface of BCP scaffolds could modulate DC adhesion morphology and spreading, exerting a modest regulatory effect on DC maturation (Figure 2A). Meanwhile, the sustained release of calcium and phosphate ions from BCP further facilitated the immunomodulatory process, and resultant immature DCs may ultimately suppress T cell activation and pro-inflammatory cytokine production [94].

Overall, DC responses to biomaterial cues are co-regulated by chemical and physical signals. The release of calcium and phosphate ions can interfere with DC biological functions, while the micro-/nano-structured surface topography modulates DC adhesion, morphology and spreading. These factors regulate DC maturation status, which in turn tunes T cell adaptive immune responses and may ultimately affect osteogenic outcomes.

#### Neutrophils

Surface micropatterning has emerged as a promising strategy to modulate neutrophil recruitment and spreading behavior. Sun *et al.* fabricated micro-/nano-groove structures with varying widths on zirconia surfaces using femtosecond laser technology and subsequently implanted these constructs into rats for *in vivo* biological evaluation. The results showed that wider grooves exerted anti-inflammatory and osteoimmunomodulatory effects. Specifically, 30 μm grooves reduced neutrophil infiltration and facilitated the phenotypic transition of macrophages from the pro-inflammatory M1 phenotype toward the anti-inflammatory M2 phenotype, compared with the narrower 6 μm grooves. This phenotypic shift facilitated the establishment of an anti-inflammatory microenvironment conducive to tissue repair. Notably, the widest groove width elicited the weakest inflammatory response, while enhancing cell adhesion and migration [95].

Surface structural features orchestrate a cascade of immune events, including neutrophil infiltration, DC maturation and T cell activation, ultimately driving M2 macrophage polarization. Notably, these effects are context-dependent and arise from synergistic physical cues rather than individual surface parameters. Therefore, rational implant design requires comprehensive evaluation of hierarchical architecture, mechanical stiffness, pore geometry and size distribution.

### Surface pore size and porosity

Porosity, defined as the fraction of void volume within a solid, can be described by parameters such as pore size, size distribution, interconnectivity and overall pore architecture [96]. In porous implants and scaffolds, these structural features first modulate the recruitment and polarization of immune cells, and further modulate osteoconduction, as well as vascular and soft-tissue ingrowth. Increased porosity has often been associated with improved cell infiltration, nutrient and oxygen transport, and elevated osteogenic differentiation. However, excessive porosity may compromise mechanical integrity and alter the local inflammatory response. Thus, there is no universal optimal parameter, and pore size and porosity need to be tuned to balance mass transport, mechanical stability and context-specific immunomodulation.

#### Macrophages

Small micropores within an implant can restrict the transport of nutrients and oxygen, thereby potentially creating a locally hypoxic microenvironment. Under excessive or persistent hypoxia, macrophages tend to upregulate pro-inflammatory markers, which may amplify inflammatory responses, facilitate granulation tissue formation and impede osteoblast infiltration. These alterations may collectively impair bone regeneration and may lead to implant failure [52]. However, hypoxia is not uniformly detrimental. Moderate hypoxic signaling can stabilize hypoxia-inducible factors (HIFs), which in turn promote macrophage-derived VEGF secretion and support vascularization, a process beneficial to bone healing [1]. These observations suggest that pore size and porosity should not be optimized simply to maximize oxygen diffusion, but rather to balance mass transport with the transient pro-angiogenic effects of early hypoxic signaling.

**Figure 4:**
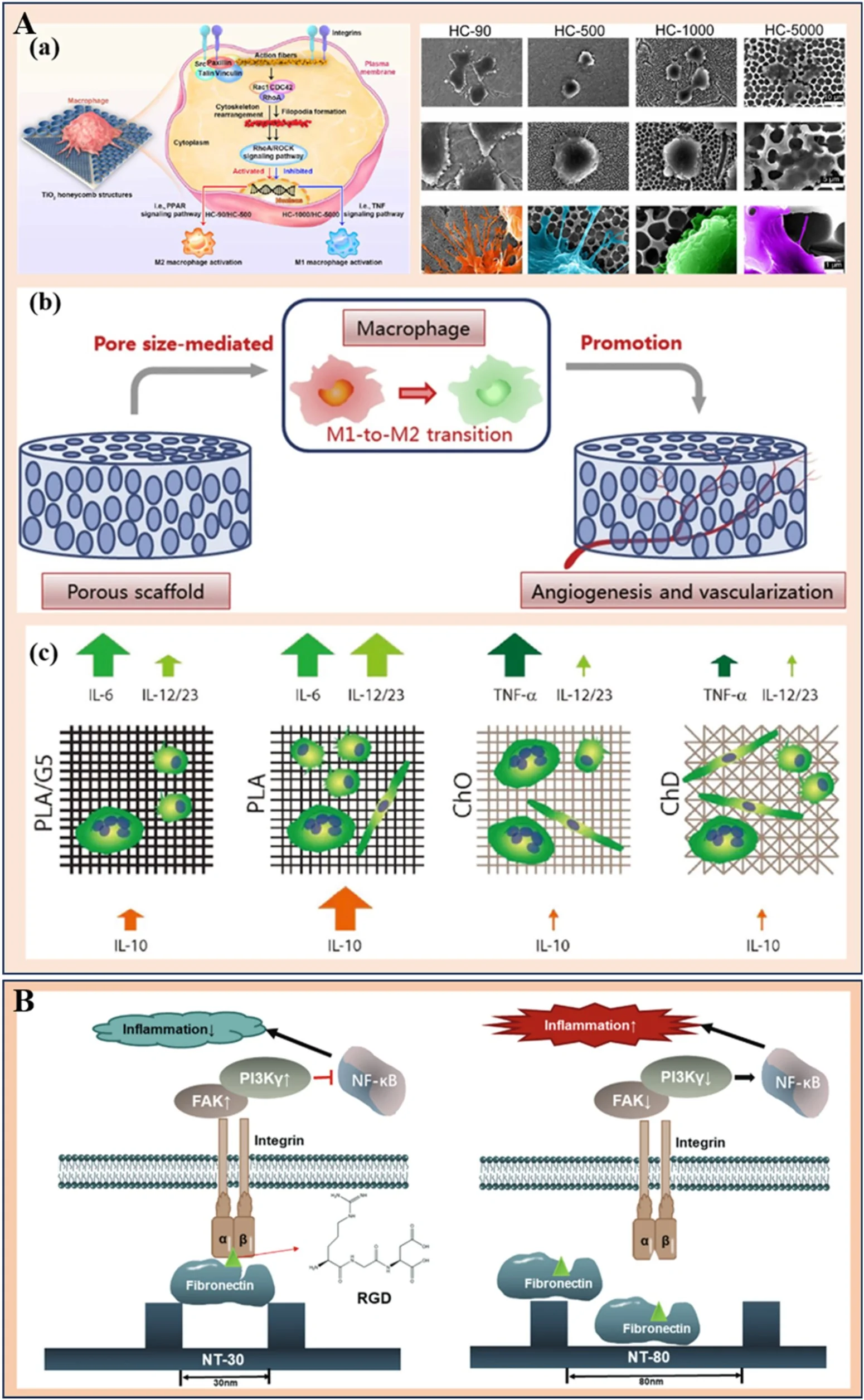
Material surface porosity and pore size affect immune cell mechanisms. (**A**) (a) Effect of surface nanostructures on macrophage polarization. Reproduced with permission from Ref. [114], Copyright 2021, Science. (b) Effect of surface micrometer structure on macrophage polarization. Reproduced with permission from Ref. [99], Copyright 2020, Elsevier. (c) Effect of scaffold structure on macrophage polarization. Reproduced with permission from Ref. [104], Copyright 2014, Elsevier. (**B**) NT30 nanotubes induce macrophage anti-inflammatory differentiation via the FAK-PI3K signaling pathway: schematic representation of the size-limiting effect of nanostructures on fibronectin-induced macrophage inflammation. Reproduced with permission from Ref. [107], Copyright 2021, Wiley.

Pore size and porosity can regulate macrophage morphology and thereby modulate their functional polarization. Evidence from cryogel systems suggests that smaller pore sizes combined with lower porosity limited M2 polarization of macrophages *in vitro*, while cryogels with larger pore sizes or higher porosity could induce M2 polarization *in vivo* [97]. Notably, this correlation is closely dependent on the material system and experimental conditions, and should not be generalized as a fixed pore size threshold for all scaffold systems. This polarization process involves transcriptional regulators such as PPAR-γ, STAT6, NF-κB and STAT1, which may compete for DNA binding sites to fine-tune M2-associated gene expression. Thus, adequate physical space, conferred by larger pores or higher porosity, appears beneficial for promoting M2 polarization and exerting anti-inflammatory effects in physiological settings [98]. For example, Yin *et al.* fabricated collagen/chitosan (Col–Ch) scaffolds with pore sizes of 160 and 360 μm. Although macrophages on both scaffolds exhibited an M1-like phenotype on day 1, the 360 μm scaffold promoted a more pronounced transition toward an M2-like state and enhanced angiogenesis both *in vitro* and *in vivo* for 3 days (Figure 4A-b) [99]. Similarly, Garg *et al.* [100] engineered electrospun polydioxanone (PDO) scaffolds with different fiber diameters, pore sizes, and porosities and found that macrophages cultured on large-pore scaffolds displayed a more spread morphology and a more M2-like phenotype, whereas small-pore scaffolds increased M1-associated marker expression. Consistent with these results, collagen/nano-hydroxyapatite scaffolds with larger lamellar spacing facilitated cell attachment and spreading, promoted timely M1 to M2 transition, and supported early angiogenesis [101]. Similarly, mineralized collagen scaffolds with micropores of approximately 84 μm and 70–80% porosity enhanced M2-like polarization together with increased TGF-β and VEGF expression [102].

Nevertheless, the relationship between pore size and macrophage phenotype is non-linear and tightly coupled with scaffold porosity and mechanical stiffness. Jiang *et al.* [103] reported that soft substrates with small pores (30 μm) favored M1-like phenotype, whereas stiff substrates with large pores (80 μm) promoted M2-like phenotype. The important role of pore geometry further supports this multi-factorial dependency, as Almeida *et al.* [104] reported that chitosan scaffolds with larger orthogonal pores (600 μm) induced a stronger pro-inflammatory response than those with smaller diagonal pores (380 μm), as shown in Figure 4A-c. Similarly, architectural constraints can shift optimal pore ranges, as Tylek *et al.* [105] found that within a box-pore scaffold system, smaller pores (40 μm) were more effective than larger pores in promoting human macrophage spreading and M2-like polarization. Collectively, these studies confirm that nominal pore size alone is insufficient to predict immune outcomes, and its effects must be interpreted in conjunction with stiffness, geometry and material chemistry. Representative studies and their major variables are summarized in Table 1.

**Table 1:** Effect of pore size and porosity on macrophage polarization

Material	Model	Main outcome	Key variables	Ref.
Cryogel (Small vs. Large pores)	*In vivo*	Larger pores favored M2-like polarization *in vivo*	Effect depends on mass transport/infiltration	[97]
Col-Ch scaffold (160 vs. 360 μm)	*In vitro*/*in vivo*	360 μm promoted faster M1 to M2 transition; angiogenesis	Time-dependent effect; chemistry plays a role	[99]
Electrospun PDO (variable pores)	*In vitro*	Large pores → M2-like; small pores → M1 markers	Fiber diameter; porosity changed simultaneously	[100]
Soft vs. Stiff substrates (30 vs. 80 μm)	*In vitro*	Stiff + large pores → M2; Soft + small pores → M1	Stiffness is a major confounder	[103]
Col/nHA scaffold (Lamellar spacing)	*In vitro*/*vivo*	Larger spacing → timely M1 to M2 transition	Architecture affects adhesion; diffusion	[101]
Mineralized collagen (∼85 μm, 70–80% porosity)	*In vitro*/*vivo*	Enhanced M2 polarization; TGF-β/VEGF expression	Pore size; porosity co-varied	[102]
Chitosan (380 vs. 600 μm, diagonal vs. orthogonal)	*In vitro*	Larger orthogonal pores induced stronger inflammation	Pore geometry > size	[104]
Box-pore scaffold (40–100 μm)	*Human macrophage*	40 μm most effective for M2-like polarization	Human-specific; pore-shape constraints matter	[105]

Discrepancies among studies may result from three major confounding factors: differences in material systems and surface chemistry; heterogeneity in *in vitro* and *in vivo* models; and coupling effects between pore size/porosity, material stiffness and topography.

The titanium dioxide (TiO_2_) nanotubes provide a well-controllable platform for investigating how nanoscale architecture regulates macrophage behavior and osseointegration-related immune responses [106]. Qi *et al.* [107] generated TiO_2_ nanotubes with diameters of 30, 80 and 150 nm (Figure 4B) and found that the 30 nm nanotubes exhibited stronger anti-inflammatory effects. Several studies have associated TiO_2_ nanotubes with reduced inflammatory activation and improved immunomodulatory performance [108, 109]. This concept is further supported by Ma *et al.* [110], who reported that nanostructured titanium surfaces enhanced implant osseointegration through macrophage-polarization-mediated immunomodulation. Similarly, Wang *et al.* [111] reported that nanostructured titanium regulated osseointegration by modulating macrophage polarization within an osteogenic microenvironment, further highlighting the coupling between nanoscale architecture, immune response, and bone formation.

Nevertheless, accumulating evidence has also identified beneficial effects induced by larger-diameter nanotubes. Xu *et al.* [112] prepared nanotubes with diameters of 60, 90 and 140 nm and found that although TiO_2_ nanotubes generally promoted anti-inflammatory polarization, larger diameters were more effective at reducing pro-inflammatory cytokine release and supporting an endothelial-compatible microenvironment. Bai *et al.* [113] reported that TiO_2_ nanotube arrays promoted macrophage polarization toward the M2-like phenotype and identified 15 nm as the optimal diameter for immunomodulation-associated osteogenesis under their experimental conditions. In contrast, Zhu *et al.* [114] reported that 90 nm performed best within their tested range, as shown in Figure 4A-a, whereas Xu *et al.* [112] highlighted the advantages of 140 nm surfaces for constructing a more anti-inflammatory microenvironment. Importantly, such diameter-dependent effects have also been observed *in vivo*. Wang *et al.* reported in a minipig model that TiO_2_ nanotubes of varying diameters (30, 70 and 100 nm) differentially regulated peri-implant gene expression and osseointegration. This finding indicates that the diameter-dependent rules of macrophage polarization and immunomodulation identified in simplified *in vitro* systems cannot be directly translated to the complex *in vivo* peri-implant microenvironment that couples immune regulation and bone osseointegration [115]. Consistent with this view, Zhang *et al.* [116] reported that highly ordered large-diameter TiO_2_ (100–200 nm) nanotube arrays supported cell proliferation and bone-forming functionality, suggesting that dimensions favorable for osteogenic performance may differ from inflammatory activation.

Therefore, the ‘optimal’ nanotube diameter is highly dependent on the research objectives, which cover multiple biological events including platelet activation, fibrin organization, macrophage polarization, endothelial compatibility, osteogenic differentiation or *in vivo* osseointegration. Bai *et al.* [113] also showed that 15 nm nanotubes could attenuate platelet activation, promote M2-like polarization and modulate fibrin fiber organization under dynamic conditions, suggesting that smaller nanotubes may be particularly advantageous for early blood–material interactions and inflammatory modulation. By contrast, larger nanotubes may support cell elongation, cytoskeletal remodeling, or secondary reparative processes. Mechanistically, such effects likely involve shape-dependent signaling and cytoskeletal tension. As reported by Fu *et al.* [117], TiO_2_ nanostructured surfaces induced M2c polarization of inflammatory monocytes via cytoskeletal rearrangement. Representative studies on TiO_2_ nanostructured titanium dimensions are summarized in Table 2.

**Table 2:** Effect of TiO_2_ nanotube diameter on immune and bone responses

Material	Model	Main outcome	Key variables	Ref.
TiO_2_ NTs (30, 80, 150 nm)	*In vitro*	30 nm showed stronger anti-inflammatory effects	Favorable under specific *in vitro* conditions	[107]
TiO_2_ NTs (15, 60, 120 nm)	*In vitro*	15 nm favored M2 polarization; platelet compatibility	Relevant to early blood–material interaction	[113]
TiO_2_ honeycomb (90 nm vs. Micro)	*In vitro*	90 nm nanoscale → M2 polarization	Nanoscale > microscale in this study	[114]
TiO_2_ NTs (60, 90, 140 nm)	*In vitro*	Larger diameters (140 nm) reduced cytokines; supported endothelium	Endpoint extends beyond macrophage phenotype	[112]
Nanostructured Ti (general)	*In vitro*/*in vivo*	Improved osseointegration via macrophage polarization (30 nm)	Supports immune-osseointegration coupling	[110]
Nanostructured Ti (osteogenic env.)	*In vivo*	Regulated osseointegration via macrophage polarization (30 nm)	Complex internal immune environment	[111]
TiO_2_ nanostructured surface	*In vitro*	M2c polarization required cytoskeletal rearrangement (30 nm)	Mechanistic insight: shape-dependent signaling	[117]
TiO_2_ NTs (various)	*In vivo*	Diameter affected peri-implant gene expression; osseointegration (70 nm)	Large animal model; not macrophage-specific	[115]
Large-diameter TiO_2_ NTs	*In vitro*	Supported proliferation; bone-forming functionality (100–200 nm)	Osteogenic *vs.* immune optimization may differ	[116]

Discrepancies among studies presented in this table mainly arise from two core confounding factors: the coupling effect of nanotube diameter with material stiffness and topological structure; heterogeneity between *in vitro* models (cell origin, culture system, detection time point) and *in vivo* models (animal species, implantation site, observation period).

In summary, pore size, porosity and pore architecture are important factors that regulate macrophage behavior and the osteoimmune microenvironment. However, their effects are highly context-dependent and cannot be attributed to a single universal design principle. In general, larger pores and higher porosity facilitate cell infiltration, mass transport and M2-like polarization, whereas nanoscale architectures frequently attenuate inflammatory activation and favor pro-regenerative immune responses. However, these trends are not consistent across all implant materials. Such inconsistent biological outcomes can be attributed to multiple material properties, including chemical composition, hardness, pore geometry, interconnectivity, permeability and degradation behavior, as well as distinct host biological responses. Moreover, the simplified *in vitro* experimental procedures cannot fully replicate the complex biological effects observed *in vivo*. Therefore, rather than being limited to one ‘optimal’ pore size or nanotube diameter, future biomaterial design should be supported by standardized material characterization and specific biological processes.

#### Neutrophils

Beyond macrophages, other innate immune cells, including neutrophils and monocytes, are sensitive to scaffold porosity and pore size. Liu *et al.* [118] reported that microporous annealed particle (MAP) scaffolds with pore sizes of 40 and 130 μm promoted enhanced neutrophil infiltration compared with 70 μm scaffolds, whereas the 70 μm scaffolds preferentially recruited monocytes, which serve as pivotal immunomodulatory and antigen-presenting cells. These findings suggest that surface microtopography may influence subsequent immune regulation primarily through modulation of immune cell recruitment dynamics rather than direct phenotypic instruction. However, these results are mainly obtained from *in vitro* or skin wound models [118], and their translational relevance to bone regeneration remains to be established.

#### T cells

T cells sense nanoscale topographic cues through the formation of actin-rich microvilli. Nanoporous surfaces facilitate microvilli formation and reprogram T cell gene expression to enhance activation. Mechanistically, confinement within approximately 200 nm pores induces spatial segregation of CD45 phosphatases and T cell receptors (TCRs) at microvilli tips, promoting the assembly of TCR nanoclusters into signaling hotspots. This spatial organization supports sustained and enhanced T cell activation even in the absence of TCR agonists [119], highlighting a ligand-independent mechanism by which physical nanoconfinement modulates T cell signaling and fate.

In addition to nanoscale effects, microscale architectural cues also modulate T cell responses. For instance, 70 μm MAP scaffolds were associated with promotion of a pro-repair, IgG1-biased T helper 2 cell (Th2) phenotype [118]. Collectively, these studies indicate that both nano- and microscale surface features play important roles in shaping T cell activation and directing the immune microenvironment toward a pro-regenerative, anti-inflammatory state.

#### Dendritic cells

The regulatory effects of pore size and porosity on DC behavior remain poorly elucidated. In an *in vivo* study, PLG scaffolds were implanted subcutaneously into the dorsal region of mice and harvested 7 days post-implantation. Compared with scaffolds possessing approximately 99% open surface pores, those with a lower effective open surface porosity of about 90.4% exhibited enhanced DC enrichment. In contrast, pore sizes larger than 10 μm modulated total cell infiltration but exerted marginal effects on DC recruitment [120]. Similarly, highly microporous (77.6 ± 2.50% porosity), low-stiffness scaffolds fabricated via low-temperature printing improved mineralization capacity but concomitantly induced pro-inflammatory DC activation [121]. Chen *et al.* [122] further reported that 40 μm pores enhanced both DC recruitment and sustained activation *in vivo*, indicating that pore size is a major modulator of DC distribution and functional status. These observations collectively suggest that the mechanical properties of small pores, including surface stiffness, indirectly influence DC recruitment and maturation.

### Surface roughness and hydrophilicity

Surface architecture fundamentally determines surface roughness and hydrophilicity, two critical physical properties that synergistically modulate protein adsorption and subsequent cellular responses. These physical properties not only modulate initial protein deposition but also regulate the recruitment and behavior of immune cells, thereby contributing to the formation of the immunomodulatory microenvironment.

#### Macrophages

**Figure 5:**
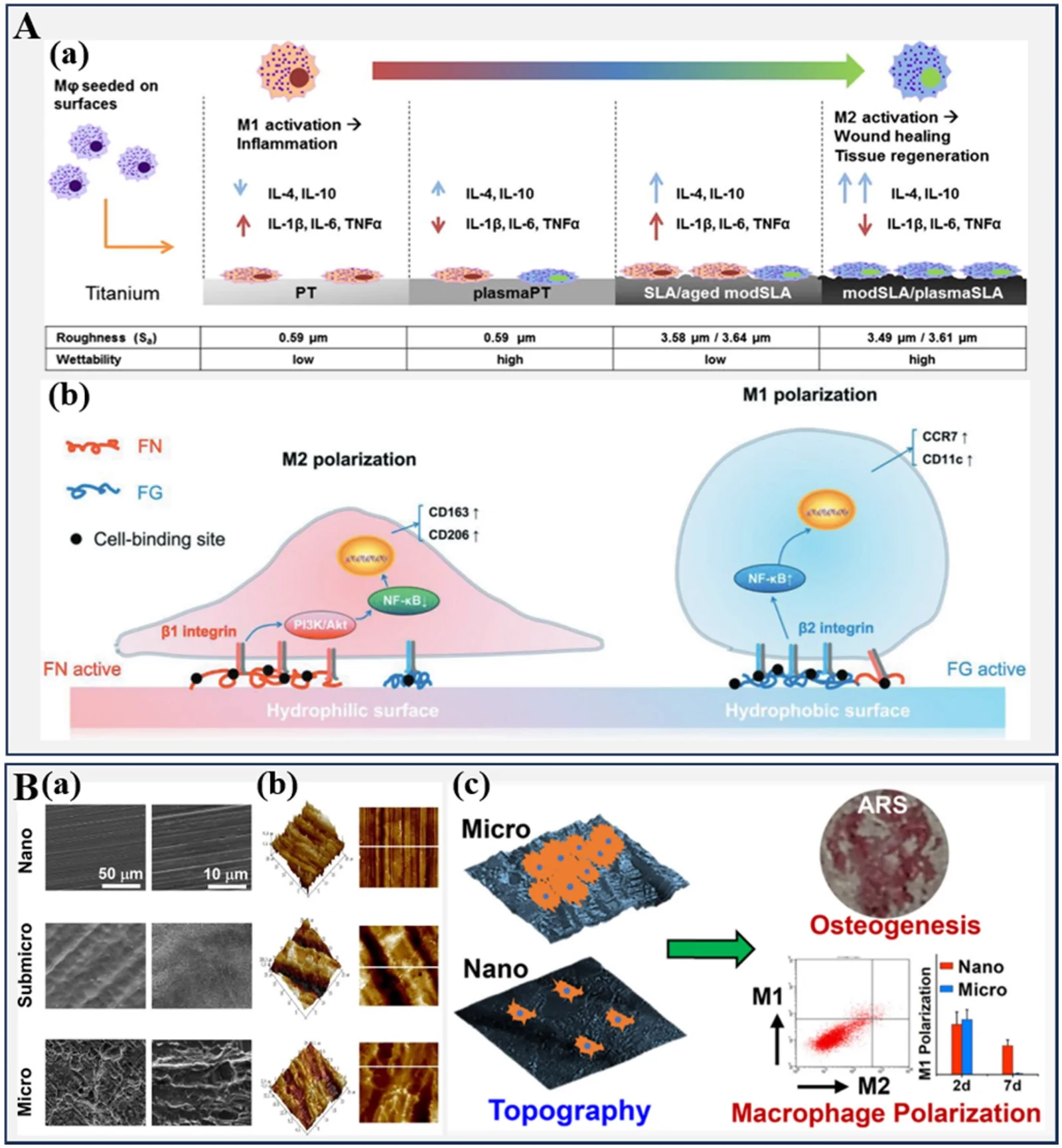
Material surface hydrophilicity and roughness affect macrophage mechanisms. (**A**) (a) Effect of surface roughness on macrophage polarization. Reproduced with permission from Ref. [125], Copyright 2016, Elsevier. (b) Effect of surface wettability on macrophage polarization. Reproduced with permission from Ref. [123], Copyright 2018, Wiley. (**B**) (a) SEM images of different textured surfaces. (b) AFM images and the line scans of surfaces. (c) Surface microroughness enhanced protein adsorption and stimulated anti-inflammatory responses. Reproduced with permission from Ref. [129], Copyright 2020, ACS.

Surface roughness and hydrophilicity act as important modulators of macrophage polarization. Lv *et al*. [123] proposed an immunomodulatory mechanism in which hydrophilic surfaces promoted M2 polarization via integrin β1 binding to fibronectin (FN), whereas hydrophobic surfaces drove M1 polarization through integrin β2 interactions with fibrinogen (FG), as illustrated in Figure 5A-b. Kai *et al*. [124] designed nanowires Ti (NW-Ti) flakes with hydrophilic properties, which enhanced M2 macrophage aggregation via integrin α5-mediated cell spreading and reduced FG-integrin αM interactions. To clarify the independent and synergistic effects of surface hydrophilicity versus roughness on macrophage polarization, Hotchkiss *et al.* systematically investigated macrophage responses to surfaces with varying roughness and wettability. Seven substrates were tested, including tissue culture control, hydrophobic and hydrophilic smooth titanium, hydrophobic and hydrophilic microrough titanium and hydrophobic and hydrophilic nano- and microrough titanium. Increased wettability consistently promoted anti-inflammatory macrophage activation more effectively than roughness alone. Notably, hydrophilic rough surfaces induced higher secretion of anti-inflammatory cytokines compared with hydrophilic smooth surfaces. These findings suggest that combining increased surface roughness with enhanced hydrophilicity can synergistically establish a favorable osteoimmune microenvironment, in which rough hydrophilic surfaces promote M2 polarization and upregulate IL-4 and IL-10 expression (Figure 5A-a) [125].

Notably, this hydrophilicity-mediated anti-inflammatory response is modulated not only by topographical features but also by diverse biomaterial surface modification strategies. Christo *et al*. [126] prepared surfaces with varying roughness, demonstrating that a 68 nm nano-Au ion-modified rough surface reduced the secretion of pro-inflammatory cytokines by primary macrophages through upregulation of matrix metallopeptidase 9 (MMP-9). Macrophage polarization is sensitive to the implant microenvironment and modulates the initial immune response [127, 128]. Cockerill *et al*. reported that zinc substrates with identical degradation kinetics but differing surface structures induced distinct inflammatory phenotypes in macrophages. Surface microroughness enhanced protein adsorption and stimulated anti-inflammatory responses (Figure 5B) [129]. Yang *et al.* found that micro-nano structured surfaces exhibited slightly lower hydrophilicity than pure nanostructured surfaces, while their increased surface roughness conferred superior anti-inflammatory properties. They suggest that M2 macrophage polarization is modulated by the interplay between surface roughness and hydrophilicity [130].

In a mechanistic investigation, Abaricia *et al*. examined Wnt signaling profiles in macrophages during pro- and anti-inflammatory polarization in response to titanium substrates with smooth, rough and hydrophilic surface characteristics. Inhibition of macrophage-derived Wnt desensitized macrophages to both physical and biological stimuli, whereas loss of macrophage-secreted Wnt impaired the recruitment of MSCs and T cells to titanium implants *in vivo*. Biomaterial surface properties modulated the expression of Wnt ligands, receptors and related molecules, with macrophages on rough hydrophilic surfaces showing upregulation of non-canonical Wnt ligands. These findings suggest a Wnt-dependent mechanism for sensing physical cues and modulating immune cell recruitment [131].

In summary, current evidence indicates that surface roughness and hydrophilicity cooperatively regulate macrophage polarization, thereby shaping the early peri-implant osteoimmune microenvironment. In general, hydrophilic and roughened surfaces favor an anti-inflammatory, pro-regenerative macrophage phenotype via integrin-mediated protein recognition, remodeled cell spreading and potentially Wnt-dependent mechanotransductive signaling. However, several limitations should be noted. First, most evidence derives from *in vitro* macrophage models, which may not fully recapitulate the complexity of the human peri-implant immune environment. Second, the respective contributions of surface roughness, hydrophilicity, chemistry and adsorbed protein layers are often difficult to disentangle, limiting precise prediction. Third, many studies still rely on the simplified M1/M2 framework, which may overlook the dynamic heterogeneity and temporal plasticity of macrophages *in vivo*. Therefore, future studies should integrate standardized surface characterization, comprehensive *in vivo* analyses and clinically relevant models to better define how specific surface parameters modulate macrophage function and osseointegration.

#### T cells

Surface roughness and hydrophilicity are important modulators of adaptive immune responses, particularly in modulating T cell recruitment and polarization. In a study by Lucke *et al*., electrospun polylactic acid (PLA) membranes and grids with enhanced hydrophilicity did not significantly alter macrophage infiltration but increased R73^+^ T cell recruitment compared with hydrophobic surfaces. The fibrous architecture of PLA grids facilitated FBGC formation during late-stage inflammation, whereas dense, non-porous and smooth-surfaced PLA membranes failed to induce such responses, suggesting that FBGCs might preferentially participate in the degradation of rough or fibrous biomaterials. Moreover, the filamentous morphology of PLA grids promoted infiltration of inflammatory and immune cells, highlighting the interplay between microstructural cues and immune cell infiltration [132].

Complementary findings were reported by Hotchkiss *et al.*, who showed that increasing the roughness and wettability of titanium implants polarized the adaptive T cell response toward a pro-regenerative Th2 phenotype, thereby accelerating inflammation resolution and MSC recruitment. *In vivo*, rough hydrophilic titanium surfaces enriched regulatory T cell (Treg) populations and increased Th2 cell abundance in both bone marrow and spleen, suggesting that antigen-presenting cell migration or implant-derived soluble factors may mediate systemic immunomodulation [133]. Similarly, Chun *et al.* reported that hydrophilic carbon nanofiber (CNF) composites elicited minimal macrophage inflammation while supporting restrained T cell activation, in contrast to hydrophobic CNFs, which enhanced T cell responses. These observations underscore that surface physicochemical properties, including microroughness and wettability, play important roles in shaping the adaptive immune microenvironment in bone tissue [134].

In summary, rough and hydrophilic surfaces effectively modulate T cell recruitment and polarization, favoring a pro-regenerative immune profile characterized by enhanced Th2-associated responses and suppressed pro-inflammatory activation. Nevertheless, most studies have focused on implants or simplified *in vitro*/*in vivo* models, and the generalizability of these findings in clinical settings remains unclear. Hence, the linkage between surface cues and T cell responses is still poorly defined and largely indirect, relying on antigen-presenting cells, macrophages or soluble mediators rather than direct T cell sensing of material properties. Another important limitation is that the heterogeneity and temporal dynamics of T cells are often insufficiently characterized. Therefore, future studies should employ more comprehensive immune phenotyping and clinically relevant models to clarify how surface roughness and hydrophilicity regulate adaptive immunity during bone healing and implant integration.

#### Dendritic cells

Surface hydrophilicity, rather than topographical roughness, appears to be a more important modulator of DC phenotype. Available studies suggest that DCs exhibit distinct inflammatory phenotypes on surfaces with similar roughness but differing wettability. For instance, sandblasted and acid-etched titanium (SLA) and its hydrophilic modification (modSLA) possess comparable surface roughness but induce opposite DC immune responses. Specifically, SLA surfaces promote DC maturation, whereas modSLA maintains DCs in an immature, low-inflammatory state, highlighting the indispensable role of surface wettability in regulating DC behaviors [135].

In summary, limited available evidence indicates that hydrophilicity may be a determinant in maintaining DCs in an immature, hypo-inflammatory state, thereby facilitating the establishment of an anti-inflammatory immune microenvironment. However, the current evidence relies on a small number of material systems, particularly comparisons between SLA and modSLA surfaces. Moreover, the respective roles of surface wettability, roughness, chemistry and topography have not been systematically separated. In addition, whether these DC phenotypic changes directly translate into predictable downstream T cell responses *in vivo* remains insufficiently understood.

#### Neutrophils

Neutrophils are sensitive to surface properties, particularly roughness and hydrophilicity, which regulate their activation and cytokine secretion [136, 137]. Early studies reported that adhesion to rough titanium surfaces may accelerate ROS production and may induce non-apoptotic cell death, indicating a heightened inflammatory response compared with smooth surfaces. Rough hydrophobic titanium surfaces could induce NET formation *in vitro* during the initial inflammatory phase [138]. However, hydrophilic surfaces reduced ROS generation and NET release by promoting the adsorption of serum proteins, thereby mitigating early immune activation [139].

Surface hydrophilicity also indirectly modulates downstream immune activity. Reduced NET formation on hydrophilic surfaces correlates with diminished macrophage activation in co-culture systems, underscoring an important role of neutrophil–macrophage crosstalk in modulating the local immune microenvironment. Similarly, hydrophobic modification of chitosan surfaces decreased neutrophil-driven acute inflammation and promoted wound healing, with IL-8 identified as a key mediator of neutrophil adhesion [140].

In general, rough hydrophobic surfaces promote neutrophil adhesion, ROS production and NET formation, whereas hydrophilic surfaces tend to attenuate these early inflammatory responses and may indirectly influence subsequent macrophage activation. However, most studies focus on acute neutrophil responses in simplified *in vitro* models, while the temporal evolution of neutrophil phenotypes *in vivo* remains poorly characterized. Therefore, future studies should employ dynamic and multi-cellular models to better define how neutrophils integrate surface cues and how these early events influence subsequent stages of osseointegration and tissue regeneration.

### Surface rigidity and hardness

Native tissues exhibit a wide range of stiffness, spanning from soft tissues including the brain (1–3 kPa) and muscle tissue (23–42 kPa), to intermediate tissues including blood vessel tissue (1.16–860 MPa) and tendon tissue (136–820 MPa), and up to rigid cortical bone (15–40 GPa). Mimicking tissue-specific stiffness in biomaterials to recapitulate the physiological mechanical context of immune cells has emerged as a critical strategy for modulating the osteoimmune microenvironment [141]. Stiffness exerts a biphasic effect on MSC osteogenic differentiation, whereby substrates below 20 kPa and above 60 kPa (e.g. 80, 90, 190 kPa) both promote osteogenesis, with higher stiffness inducing a stellate morphology characteristic of osteoblasts [142]. An important question remains whether bone-associated immune cells exhibit similar biphasic or stiffness-dependent responses, warranting further investigation into the mechano-immunological crosstalk during bone tissue healing.

#### Macrophages

**Figure 6:**
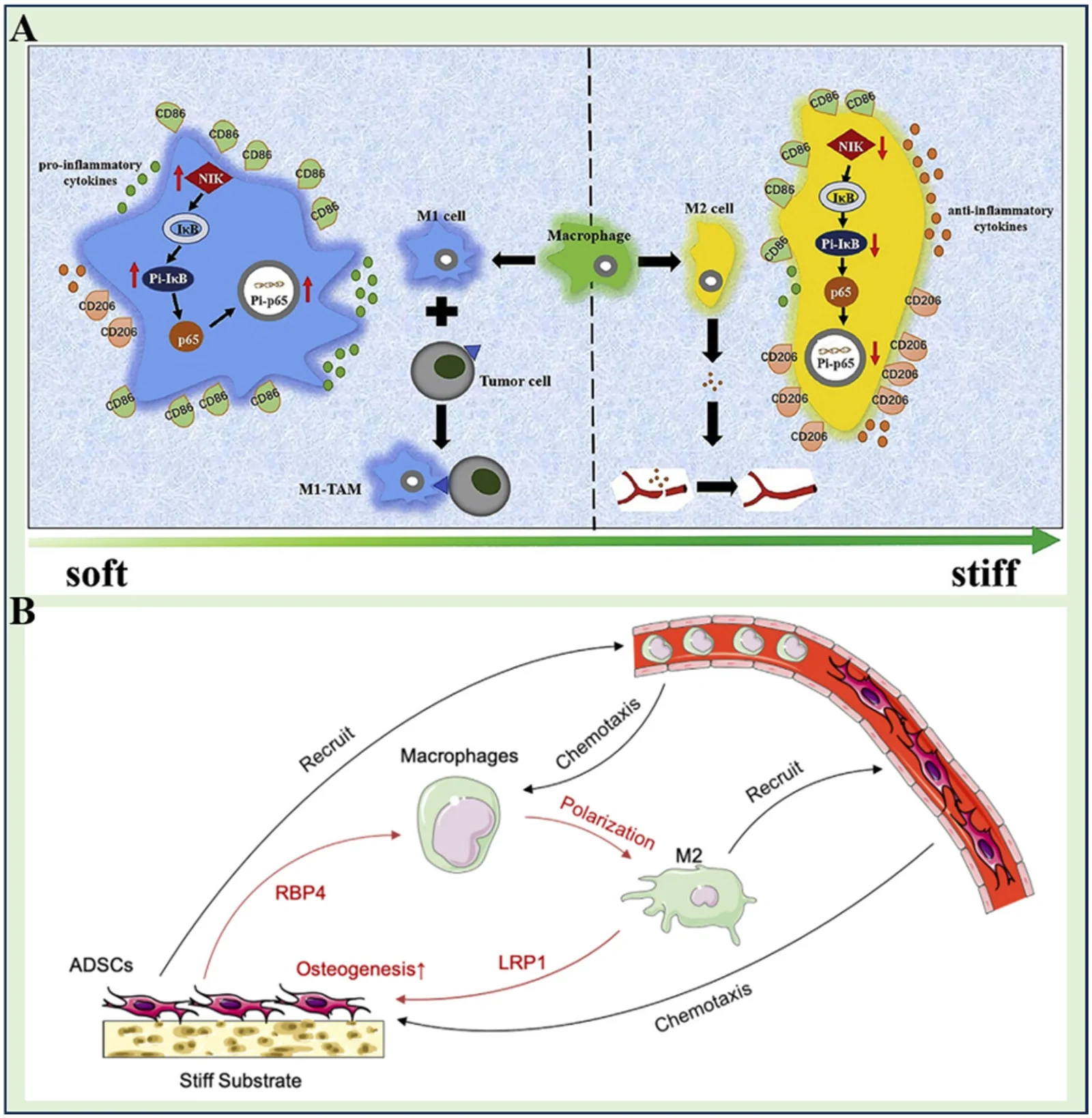
Surface stiffness affects macrophage mechanisms. (**A**) Macrophage polarization can be influenced by sensing the stiffness of the ECM. Reproduced with permission from Ref. [143], Copyright 2018, Elsevier. (**B**) Schematic illustration of the crosstalk between ADSCs and macrophages under the influence of stiff biomaterials. Reproduced with permission from Ref. [150], Copyright 2023, Frontiers.

Cellular responses are influenced by mechanical cues, with macrophages exhibiting marked phenotypic plasticity in response to substrate stiffness (Figure 6A). Accumulating evidence indicates that substrate stiffness modulates macrophage polarization, in which high-stiffness substrates generally promote pro-inflammatory M1 phenotypes, whereas low-stiffness environments favor anti-inflammatory M2 polarization [143, 144]. For example, macrophages cultured on low-stiffness TG-gels preferentially adopt an M2 phenotype [145]. Similarly, hydrogels with a stiffness of 60 kPa can promote bone regeneration through the synergistic enhancement of both osteogenic differentiation and M2 macrophage polarization [146].

In one study, hydrogels with stiffness values of 2, 32 and 62 kPa were evaluated, revealing that matrices mimicking collagen fibers (32 kPa) were associated with increased M1-like markers via NOS-mediated NF-κB signaling, whereas osteoid-like stiffness (62 kPa) was linked to M2-like markers. This biphasic trend is consistent when considering the specific stiffness ranges examined [147]. Further supporting this biphasic trend, Sridharan *et al.* observed that polyacrylamide gels with high stiffness (323 kPa) were associated with pro-inflammatory phenotypes and reduced phagocytic activity, whereas soft (11 kPa) and moderately stiff (88 kPa) gels were linked to anti-inflammatory, highly phagocytic macrophages [148].

The mechanosensitivity of macrophages may arise from stiffness-dependent changes in protein adsorption and cell adhesion dynamics [149]. In addition, substrate stiffness influences macrophage behavior through intercellular crosstalk. For example, Liu *et al.* reported that adipose-derived stem cells (ADSCs) cultured on stiffer substrates (64 kPa versus 0.2 kPa) secreted immunomodulatory proteins (LBP, RBP4), which promoted M2-like polarization and chemotaxis of macrophages. Reciprocally, M2 macrophages secreted ECM remodeling proteins, including LRP1, which facilitated ADSC osteogenic differentiation and recruitment of local stem cells (Figure 6B) [150].

Mechanosensitive regulation also extends to foreign body responses, an important factor influencing long-term implant osseointegration. Meli *et al.* [151] reported that increased hydrogel stiffness not only amplified inflammatory activation of porcine macrophages by more than twofold but also promoted their fusion into FBGCs. Conversely, adhesion to softer hydrogels attenuated inflammatory responses, correlating with reduced expression and nuclear localization of YAP, a key mechanosensitive regulator of macrophage inflammation. Subsequent *in vivo* experiments showed that soft substrates reduced both inflammatory marker expression and YAP activity in the peri-implant macrophages relative to stiffer substrates [152].

In summary, these studies covered an elastic modulus range of approximately 1–323 kPa. Collectively, the findings consistently indicate that substrate stiffness modulates the polarization state of macrophages and subsequent osteogenic outcomes. The divergent stiffness-dependent macrophage behaviors are largely influenced by differences in model systems (2D vs. 3D culture), material degradability and dynamic mechanical changes during hematoma remodeling. Accumulating evidence consistently supports that low-to-moderate stiffness (10–88 kPa) generally favors M2-like macrophage polarization in 2D *in vitro* models, while the *in vivo* effects are closely coupled to scaffold architecture and material degradation, with no universal optimal stiffness threshold identified to date.

#### T cells

**Figure 7:**
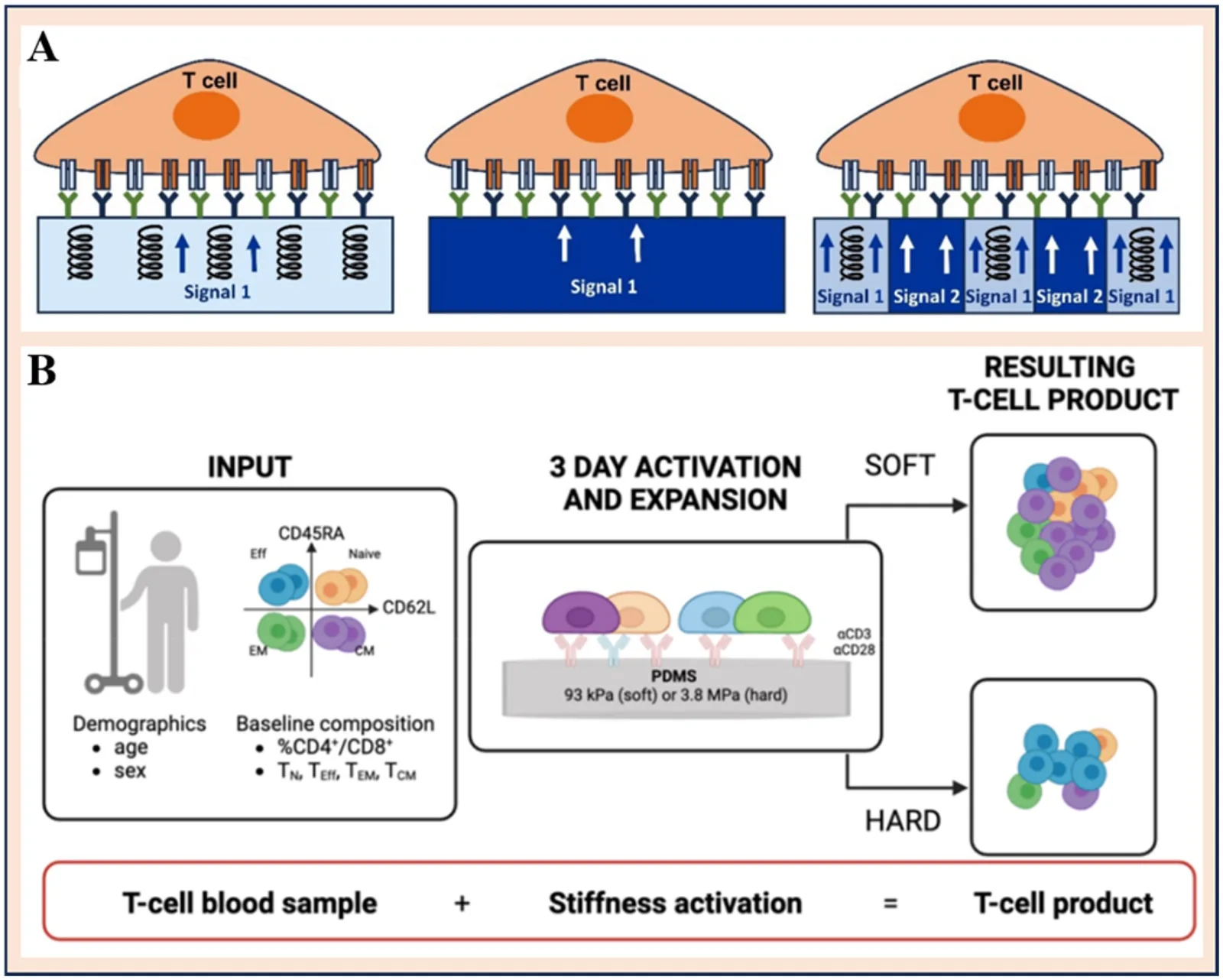
T cell responses to surface stiffness. (**A**) Two activation schemes of the microenvironment, soft and stiff, enable mechanical patterning to provide distinct mechanical signals to individual T cells at the same time. Reproduced with permission from Ref. [156], Copyright 2012, Oxford. (**B**) Schematic of the donor characteristics on mechanosensitive swelling. Reproduced with permission from Ref. [160], Copyright 2020, Elsevier.

T cell morphology and early activation are sensitive to substrate stiffness. Stiffer hydrogels (50.6 ± 15.1 kPa) promote increased IL-2 secretion while restricting T cell expansion efficiency [153]. Yuan *et al.* [154] reported that T cell proliferation and IL-2 production exhibited a biphasic response to substrate stiffness, and this behavior was modulated by the concentration of activating antibodies and abolished when cellular contraction was inhibited. Notably, T cell mechanosensitivity peaks at stiffness magnitudes comparable to those of antigen-presenting cells (APCs, tens of kPa), with T cell responsiveness declining at higher stiffness levels [155]. This peak-response characteristic explains the seemingly inconsistent findings between studies; for example, O’Connor *et al.* reported that soft substrates (Young’s modulus < 100 kPa) elicited approximately fourfold higher IL-2 production and proliferation of human CD4^+^ and CD8^+^ T cells relative to stiff substrates (>2 MPa) (Figure 7A) [156].

To better recapitulate the mechanical heterogeneity of native *in vivo* microenvironments, T cells were cultured on alternating soft and stiff regions. Notably, T cells exhibited immune responses similar to those on uniformly soft substrates, rather than generating averaged mechanical signal integration, indicating nonlinear mechanosensing behaviors [157]. Regulatory T cell induction was also strongly stiffness-dependent, in which softer substrates (∼100 kPa) were associated with enhanced Treg differentiation relative to stiffer substrates (∼3 MPa) [158]. Treg induction on stiff substrates involved elevated oxidative phosphorylation (OXPHOS), suggesting that mechanosensitive Treg differentiation is accompanied by metabolic reprogramming [159]. In three-dimensional culture systems, higher substrate stiffness (3.8 MPa) was linked to increased T cell activation, proliferation and migration compared with softer environments (93 kPa) (Figure 7B) [160]. Collectively, these findings indicate that T cells, similar to macrophages, integrate substrate stiffness cues in a nonlinear, context-dependent manner, providing new insights for the design of immunomodulatory biomaterials in bone regeneration.

#### Dendritic cells

DC behavior is sensitive to both matrix degradability and stiffness. In the study by Choi *et al*., the hydrogels were classified into low (0.5 kPa), medium (0.8 kPa) and high (2 kPa) stiffness, enabling systematic investigation of stiffness-dependent effects on DC behavior. Their results showed that soft, proteolytically degradable substrates favored DC proliferation, whereas stiffer hydrogels tended to enhance DC differentiation and maturation [161]. When cultured on polyacrylamide substrates of varying stiffness (2, 12 and 50 kPa), immature DCs exhibited altered C-type lectin expression, which modulated antigen internalization [162]. Furthermore, substrate stiffness affects β2 integrin expression and podosome formation in immature DCs, highlighting its role in modulating DC adhesion and mechanosensing. Collectively, these findings suggest that both hydrogel degradability and mechanical properties are important modulators of DC behavior, with potential implications for designing immunomodulatory scaffolds in bone regeneration.

#### Neutrophils

The effect of substrate stiffness on neutrophils is largely reflected in their infiltration and recruitment dynamics. Sadtler *et al.* systematically explored the role of material stiffness in neutrophil recruitment using poly(ethylene glycol) (PEG) hydrogels with tunable mechanical properties (shear storage modulus ranging from 0.5 to 10 kPa). They reported that neutrophil infiltration was dependent on both stiffness and pore size. Notably, stiffer gels (5–10 kPa) promoted greater neutrophil infiltration than softer gels (0.5–2 kPa), maintaining dense neutrophil presence for 1–3 weeks post-injury, but this did not significantly alter fibrosis around the material, thereby highlighting a complex, non-linear relationship between acute inflammation and long-term fibrotic outcomes [163].

Overall, softer hydrogels generally favor immune tolerance or wound-healing phenotypes, whereas stiffer hydrogels tend to promote immune activation. Collectively, these studies indicate that matrix stiffness can modulate multiple immune cell populations, including neutrophils, macrophages, T cells and DCs, and that mechanical cues play an important role in shaping the immune microenvironment. Notably, most of these studies were performed in non-bone models, and their conclusions should be referenced with caution when applied to bone regeneration research.

Importantly, all observations summarized herein are derived from the published studies. The immunomodulatory effects of each physical parameter are context-dependent, which are modulated by material chemistry, experimental model systems and co-occurring surface modifications. Furthermore, the translational potential of these physical regulatory strategies requires further systematic validation in multicellular dynamic systems and high-temporal-resolution *in vivo* models.

## Conclusions and outlook

This review emphasizes the notable role of the early hematoma stage in bone defect healing. Cumulative evidence suggests that the hematoma is not merely a transient blood clot, but a biologically active, dynamically regulated and stage-specific osteoimmune niche during the earliest phase of bone healing. Within this microenvironment, neutrophils, macrophages, DCs, T cells and B cells interact with the fibrin network, cytokines and physicochemical cues on biomaterial surfaces to collectively shape immune responses. Implant surface properties further modulate these early events, thereby influencing immune cell phenotypes, hematoma maturation and subsequent bone regenerative outcomes.

However, direct evidence illustrating how biomaterial surface properties remodel hematoma composition and fibrin architecture remains relatively limited. Most current studies instead focus on downstream immune indicators, particularly macrophage polarization in simplified *in vitro* systems. Furthermore, the characterization of DCs, T cells, neutrophils and B cells in the context of biomaterial-mediated hematoma regulation remains largely insufficient. Current trends suggest that the biophysical properties of the biomaterial surface direct the evolving osteoimmune microenvironment toward either a pro-inflammatory or a pro-regenerative phenotype. However, such biological outcomes are governed by multiple confounding factors, including material chemistry, topography, mechanical properties, animal models, species differences and downstream biological responses. Therefore, future research should prioritize experimental systems with high temporal resolution and multicellular complexity that recapitulate the hematoma microenvironment. Meanwhile, the development of standardized protocols for tissue characterization and immunophenotypic analysis is important for investigating the relationship between hematoma behavior and subsequent bone formation outcomes.

From clinical and translational perspectives, future advances may depend on three core directions. First, establishing standardized multicellular biomimetic hematoma models for both physiological and pathological bone repair (e.g. osteoporosis and diabetes-associated bone healing impairment) to decouple the independent and synergistic effects of distinct material physical cues. Second, developing stage-specific immunomodulatory implants with temporal parameter matching to hematoma progression, so as to precisely guide the transition of inflammation from a defensive state to a resolutive and pro-regenerative state. Third, constructing an evidence-based implant physical parameter design database to clarify the applicable scenarios, confounding variables and translational feasibility of diverse surface modification strategies, thereby avoiding overgeneralization of conclusions derived from simplified *in vitro* assays. Given the dynamic nature of the early hematoma, artificial intelligence (AI) may serve as a promising tool to decode the complex, non-linear correlations between implant surface biophysical parameters, spatiotemporal hematoma organization, the dynamics of multiple immune cell populations and long-term bone healing outcomes. The integration of AI with *in viv*o imaging, single-cell spatial transcriptomics and high-throughput material screening may help establish a conceptual framework for next-generation bone repair strategies.
